# Programming Mn(II) coordination in self-assembling peptides amplifies mtDNA-driven STING signaling for potent antitumor immunity

**DOI:** 10.1016/j.mtbio.2026.103373

**Published:** 2026-06-18

**Authors:** Guoyu Xia, Chenyang Wang, Liyuan Peng, Weiyu Xing, Lulu Wang, Zhen Zheng

**Affiliations:** The Province and Ministry Co-sponsored Collaborative Innovation Center for Medical Epigenetics, Tianjin Key Laboratory on Technologies Enabling Development of Clinical Therapeutics and Diagnostics, School of Pharmacy, Tianjin Medical University, Tianjin, 300070, China

**Keywords:** Self-assembling peptide, Manganese nanomedicine, Metal-organic coordination, mtDNA release, cGAS–STING pathway

## Abstract

Activation of the cGAS–STING pathway is a powerful antitumor strategy, but its therapeutic performance is often constrained by insufficient cytosolic DNA cues. Here, we report a bioinspired metallopeptide nanoassembly, RKLAHE–Mn, formed through dynamic N,O-bidentate coordination between Mn(II) and a programmable hexapeptide scaffold. By leveraging specific interactions between histidine and glutamic acid, we achieve robust chelation that orchestrates the self-assembly of uniform, chain-like assemblies of spherical nanoparticles, with DFT calculations supporting a thermodynamically favorable Mn–His/Glu N,O-coordination mode. Upon internalization, RKLAHE–Mn triggers a rapid ROS burst and perturbs intracellular redox homeostasis. These cationic assemblies preferentially accumulate in mitochondria, leading to loss of mitochondrial membrane potential and promoting mitochondrial DNA (mtDNA) leakage into the cytosol. The released mtDNA, together with Mn(II)-dependent potentiation, amplifies STING signaling and elicits immunogenic cell death–associated responses. Notably, RKLAHE–Mn also potentiates STING-related innate immune signaling in dendritic cells, driving maturation with upregulated CD80, CD86, and MHC class II expression, increased secretion of IL-6, TNF-α, and CXCL10, and enhanced antigen presentation and cross-presentation. In a murine breast cancer model, RKLAHE–Mn remodels the immunosuppressive tumor microenvironment by promoting dendritic cell maturation and CD8^+^ T cell infiltration, and it significantly enhances the therapeutic efficacy of anti-PD-L1 checkpoint blockade. Collectively, this work establishes metal-coordination–guided peptide self-assembly as a versatile paradigm for converting ionic cofactors into targeted, immunostimulatory nanotherapeutics for cancer immunotherapy.

## Graphical abstract

**Figure:**
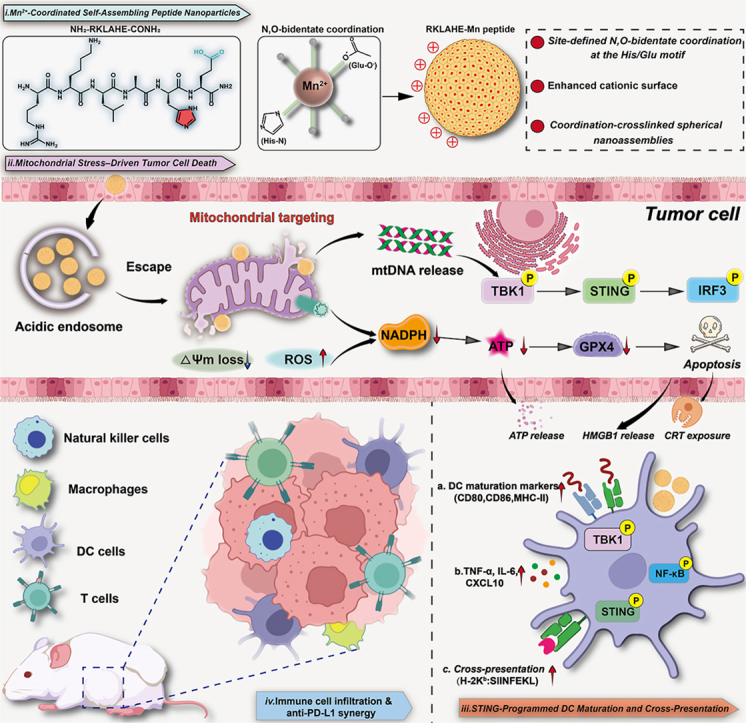

## 1 Introduction

**Scheme 1:**
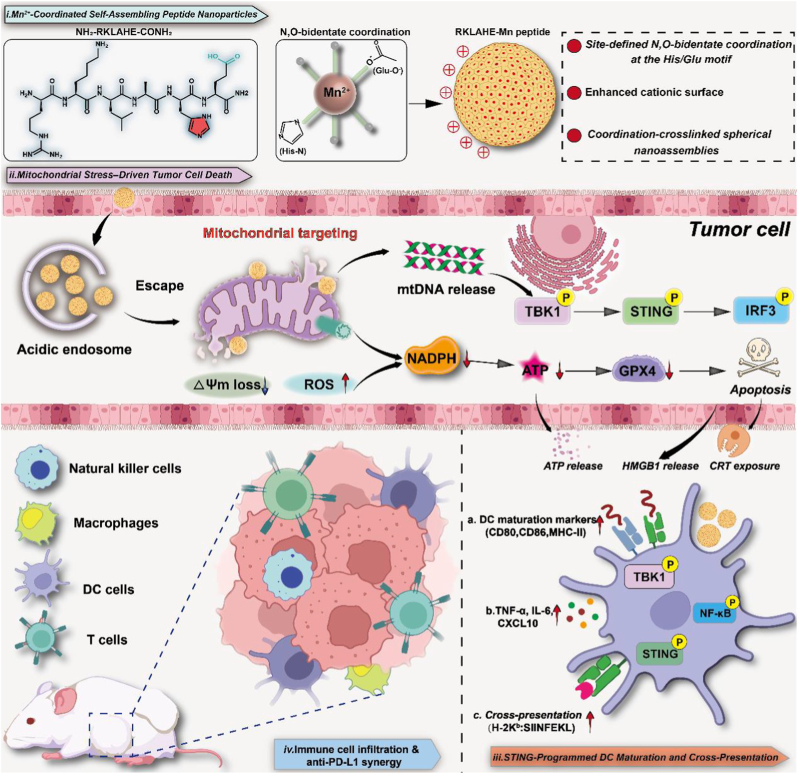
**Design rationale and proposed immunotherapeutic mechanism of RKLAHE–Mn.** (i) Mn^2+^ forms N,O-bidentate coordination with O/N donor sites from Glu (E) and His (H) within a self-assembling peptide, yielding dynamically crosslinked RKLAHE–Mn nanoassemblies. (ii) Upon mitochondrial enrichment, RKLAHE–Mn induces mitochondrial stress, leading to mitochondrial membrane potential dissipation and cytosolic mtDNA release, which subsequently activates phosphorylation of downstream STING-associated signaling proteins; concomitant redox imbalance promotes immunogenic tumor cell death. (iii) RKLAHE–Mn also activates STING-related innate immune signaling in dendritic cells, thereby promoting DC maturation and enhancing proinflammatory cytokine secretion and antigen cross-presentation. (iv) Through the cascade of tumor cell antigen release and enhanced DC antigen presentation, RKLAHE–Mn facilitates recruitment and infiltration of M1-like macrophages, DCs, CD8^+^ T cells, and NK cells in the tumor microenvironment; combination with anti–PD-L1 further alleviates immunosuppression and amplifies antitumor immune responses.

Metallopeptides, generated through the precise coordination of metal ions with programmable peptide scaffolds, represent a versatile biological strategy for integrating structural stabilization with catalytic and signaling functions [[1], [2], [3]]. Numerous archetypal systems illustrate that the synergy between metal ions and peptide backbones can confer unique biological functions[[4], [5], [6]]. For example, copper peptide–related systems exhibit multilayer regulatory roles in tissue repair and antioxidation, while metalloproteins such as hemoglobin precisely tune molecular recognition and reactivity through well-defined coordination environments[[7], [8], [9], [10]]. These observations suggest that peptide-based coordination chemistry is not only a structural construction tool but also a promising entry point for developing new biomaterials and immunomodulatory strategies. Recent studies have shown that nanomedicines can potentiate cancer therapy by reshaping the tumor microenvironment, enhancing local immunotherapy, regulating immune checkpoint-related suppression, and inducing immune-associated cell death pathways[[11], [12], [13], [14], [15]]. However, in the context of cancer immunotherapy, the use of programmable metal–peptide materials to modulate immune signaling remains insufficiently explored (see Scheme 1).

Recent findings have identified Mn^2+^ as a critical potentiators of the STING pathway. Mn^2+^enhances the sensitivity of cGAS to double-stranded DNA and directly facilitates cGAMP synthesis [16,17]. Representative Mn-STING nanoplatforms have demonstrated the therapeutic potential of Mn^2+^-assisted STING activation, but many rely on exogenous agonists or complex multicomponent designs. This highlights the need for simpler systems that couple Mn delivery with endogenous DNA release and mitochondrial stress [[18], [19], [20]]. Notably, in the absence of exogenous DNA, free Mn^2+^ typically requires millimolar concentrations to induce robust activation of the cGAS–STING pathway [21,22]. Therefore, this underscores the need for strategies that provide endogenous DNA stimuli and enable localized signal amplification. In this context, recent mitochondria-targeted manganese-based nanoplatforms have demonstrated that mitochondrial damage-mediated mtDNA release can effectively cooperate with intracellular Mn^2+^ delivery to amplify cGAS–STING signaling and sensitize tumors to anti-PD-L1 therapy [23]. Unlike nuclear DNA, which is shielded by histones, mtDNA is highly susceptible to stress-induced leakage [24,25]. Given the highly electronegative mitochondrial microenvironment, engineering self-assembling peptides with cationic basic residues or protonatable groups to modulate surface charge and promote mitochondrial tropism represents an attractive approach.

Supramolecular peptide self-assembly offers a robust strategy to overcome delivery and activation barriers in immunotherapy. Distinguished by their sequence programmability, structural modularity, and inherent biocompatibility, these peptide scaffolds can be precisely tuned for specific morphologies and surface charges [26,27]. By precisely arranging amino acids, these scaffolds can be tuned to achieve specific morphologies and surface charges; for instance, incorporating cationic or protonatable motifs facilitates interaction with anionic cellular membranes and directs intracellular trafficking[[28], [29], [30]]. Crucially, peptides can be engineered with side chains capable of metal coordination, such as imidazole or carboxylate groups. In this context, metal ions serve not merely as passive cargo, but as active structural crosslinkers that stabilize the assembly while simultaneously modulating its local electronic environment and bioactivity [31].

Building on these principles, we developed a coordination-driven metallo-peptide nanoassembly, RKLAHE-Mn (composed of the peptide Arg-Lys-Leu-Ala-His-Glu coordinated with Manganese), designed to amplify STING signaling via a mitochondrial stress mechanism. We engineered an amphiphilic peptide scaffold containing native N,O-donor sites from histidine and glutamic acid to enable dynamic bidentate coordination with Mn^2+^. This coordination strategy serves a dual purpose: driving the self-assembly of stable nanostructures and reconfiguring surface charge to promote mitochondrial tropism. We demonstrate that upon internalization, RKLAHE-Mn accumulates in mitochondria, inducing membrane potential dissipation and the leakage of mtDNA into the cytosol. This release of endogenous DNA, combined with the intracellular delivery of Mn^2+^, triggers a potent activation of the cGAS-STING axis and elicits immunogenic cell death (ICD). Furthermore, RKLAHE-Mn promotes the maturation and antigen cross-presentation capabilities of dendritic cells. In a murine breast cancer model, this cascade remodels the TME by promoting the infiltration of CD8^+^T cells, NK cells, and dendritic cells, thereby sensitizing tumors to anti-PD-L1 therapy. Collectively, this work establishes metal-coordination-guided peptide assembly as a robust strategy to convert ionic cofactors into targeted, self-amplifying immunotherapeutics.

## 2 Experimental section

### 2.1 Materials

Fmoc-Glu(OtBu)-OH, Fmoc-His(Trt)-OH, Fmoc-β-Ala-OH, Fmoc-Leu-OH, Fmoc-Lys(Boc)-OH, Fmoc-Arg(Pbf)-OH, and Rink amide MBHA resin (4-methylbenzhydrylamine resin) were purchased from Shanghai Bide Pharmatech Co., Ltd. (Shanghai, China). N,N-diisopropylethylamine (DIPEA) was purchased from Shanghai Macklin Biochemical Co., Ltd. (Shanghai, China). O-(Benzotriazol-1-yl)-N,N,N′,N'-tetramethyluronium hexafluorophosphate (HBTU) was obtained from Titan Scientific Co., Ltd. (Shanghai, China). Piperidine was purchased from Tianjin Damao Chemical Reagent Factory (Tianjin, China). Trifluoroacetic acid (TFA) was purchased from Tianjin Kemiou Chemical Reagent Co., Ltd. (Tianjin, China). Other organic solvents were purchased from Tianjin Jindong Tianzheng Fine Chemical Reagent Factory (Tianjin, China). Unless otherwise specified, chemicals were used as received.

Manganese(II) chloride tetrahydrate (MnCl_2_·4H_2_O), 4-chloro-7-nitrobenzofurazan (NBD-Cl), and IR-820 (IR820) were purchased from Shanghai Macklin Biochemical Co., Ltd. (Shanghai, China). OVA_257–264_ peptide (SIINFEKL) was purchased from Sangon Biotech Co., Ltd. (Shanghai, China). Anti–PD-L1 monoclonal antibody was purchased from Bio X Cell (Lebanon, NH, USA). MTT (3-(4,5-dimethylthiazol-2-yl)-2,5-diphenyltetrazolium bromide) was purchased from HEOWNS Biochemical Technology Co., Ltd. The ATP assay kit (S0026), NADP^+^/NADPH assay kit (S0179), Annexin V-FITC apoptosis detection kit (C1062M), TMRE mitochondrial membrane potential assay kit (C2001S), Mitochondria Deep Red dye (C1997S), Lyso-Tracker Red (C1046), and ROS detection kit (DCFH-DA, S0033S) were purchased from Beyotime Biotechnology (Shanghai, China).

Primary antibodies used in immunofluorescence and western blotting included Calreticulin Rabbit mAb (A20986), HMGB1 Rabbit mAb (A19529), TBK1/NAK Rabbit mAb (A3458), Phospho-TBK1/NAK (Ser172) Rabbit mAb (AP1026), and GPX4 Rabbit mAb (A11243) (ABclonal, Wuhan, China). Secondary antibodies for immunofluorescence included FITC-conjugated Goat anti-Rabbit IgG (H + L) (AS011) and Cy3-conjugated Goat anti-Rabbit IgG (H + L) (AS007) (ABclonal, Wuhan, China). GAPDH monoclonal antibody (60004-1-Ig), HRP-conjugated Goat anti-Rabbit IgG (H + L) (SA00001-2), and HRP-conjugated Goat anti-Mouse IgG (H + L) (SA00001-1) were purchased from Proteintech (Wuhan, China).cGAS Rabbit mAb (D3O8O), STING Rabbit mAb (D2P2F), Phospho-STING (Ser366) Rabbit mAb (D7C3S), IRF-3 Rabbit mAb (D83B9), Phospho-IRF-3 (Ser396) Rabbit mAb (4D4G), and Phospho-NF-κB p65 (Ser536) Rabbit mAb (93H1) were purchased from Cell Signaling Technology (Danvers, MA, USA). Fluorophore-conjugated antibodies for flow cytometry, including PE/Cy7-*anti*-CD11c, BV421-*anti*-CD80, APC-anti-CD86, FITC-anti-CD3, PE-anti-SIINFEKL-H-2K^b^, APC-anti-CD4, PE-anti-CD8, APC/Cy7-*anti*-CD11b, PerCP/Cy5.5-*anti*-CD45, and FITC-anti-NKp46, were purchased from BioLegend.

### 2.2 Instruments

Analytical high-performance liquid chromatography (HPLC) was performed using a Waters 2695 separation module (Waters, USA). High-resolution mass spectrometry (HRMS) was carried out on an LTQ Orbitrap XL mass spectrometer (Thermo Scientific, San Jose, CA, USA). Transmission electron microscopy (TEM) images were acquired on an HT7800 transmission electron microscope (Hitachi, Japan). Circular dichroism (CD) spectra were recorded on a J-715 spectropolarimeter (JASCO, Japan). UV–vis absorption spectra were measured using an Infinite 200 PRO microplate reader (TECAN, Switzerland).

X-ray photoelectron spectroscopy (XPS) was performed on an ESCALAB 250Xi system (Thermo Fisher Scientific, USA). X-ray diffraction (XRD) patterns were collected on an Ultima IV diffractometer (Rigaku, Japan). Fourier-transform infrared (FT-IR) spectra were obtained using a Nicolet iN10 spectrometer (Thermo Fisher Scientific, USA). *In vivo* and ex vivo fluorescence imaging was conducted on an IVIS Spectrum imaging system (PerkinElmer, USA). Flow cytometry data were acquired on a BD FACSVerse flow cytometer (BD Biosciences, USA). Confocal laser scanning microscopy was performed using an LSM 980 microscope (ZEISS, Germany).

### 2.3 Cell lines and animals

4T1 and DC2.4 cell lines were obtained from the Cell Bank of the Chinese Academy of Sciences (Shanghai, China). Cells were cultured in RPMI 1640 medium supplemented with 10% fetal bovine serum (FBS) and 1% penicillin–streptomycin at 37 °C in a humidified incubator with 5% CO_2_. Female BALB/c mice (6–8 weeks old) were purchased from SPF Biotechnology Co., Ltd. (Beijing, China) and maintained under specific pathogen-free conditions in a controlled environment (25 °C, 55 ± 5% humidity, 12 h light/12 h dark cycle). All animal procedures were conducted in accordance with relevant guidelines and were approved by the Institutional Animal Care and Use Committee of Tianjin Medical University (Approval No. TMUaMEC202300).

### 2.4 Synthesis of RKLAHE, RKLAHE–Mn^NBD^, and RKLAHE–Mn^IR820^

RKLAHE peptide was synthesized on Rink amide MBHA resin (4-methylbenzhydrylamine resin) using standard Fmoc-based solid-phase peptide synthesis (SPPS) protocols. Briefly, iterative Fmoc deprotection and amino-acid coupling were performed following conventional Fmoc chemistry. After completion of the peptide chain assembly, the terminal Fmoc group was removed to expose the free N-terminal amine.

For on-resin N-terminal labeling, the resin-bound peptide was reacted with NBD-Cl or activated IR820 under basic conditions to afford N-terminally labeled peptides. After labeling, peptides were cleaved from the resin using a cleavage cocktail of TFA/TIS/H_2_O (95:2.5:2.5, v/v/v) for 2 h. The crude products were precipitated with cold diethyl ether, collected by centrifugation, re-dissolved in appropriate solvents, and purified by preparative reverse-phase HPLC. The purified peptides were characterized by mass spectrometry to confirm the expected molecular weight, and purity was assessed by HPLC.

### 2.5 Preparation of RKLAHE–Mn peptide nanoassemblies

To generate Mn^2+^-coordinated peptide assemblies, RKLAHE was dissolved in HEPES buffer (pH 7.6). MnCl_2_·4H_2_O was then added to achieve a peptide-to-Mn^2+^ molar ratio of 1:4. The mixture was incubated overnight at 37 °C with gentle shaking protected from light to facilitate Mn^2+^ coordination with the peptide binding sites and promote the formation of Mn^2+^-reinforced peptide nanoassemblies. After incubation, the samples were purified by dialysis using a MWCO 100–500 Da dialysis membrane to remove unbound Mn^2+^ and small ions, yielding purified RKLAHE–Mn. The same coordination procedure was applied to N-terminally labeled RKLAHE (RKLAHE^NBD^ or RKLAHE^IR820^) to obtain RKLAHE–Mn^NBD^ and RKLAHE–Mn^IR820^, respectively.

### 2.6 Cellular uptake assay

4T1 cells were seeded in 12-well plates at 1 × 10^5^ cells per well and cultured overnight. Cells were then incubated with RKLAHE^NBD^ (final concentration, 50 μM) for 1 h or 4 h. After incubation, cells were washed with PBS (three times), stained with Hoechst 33342 for nuclear labeling, and imaged using an inverted fluorescence microscope.

For flow cytometry analysis, 4T1 cells were seeded under the same conditions and treated as described above. Cells were harvested by trypsinization, washed, resuspended in PBS, and analyzed by flow cytometry. The intracellular fluorescence intensity was quantified using the FITC channel.

### 2.7 Intracellular ROS measurement

4T1 cells were seeded in 12-well plates at 2 × 10^5^ cells per well and cultured overnight. After treatment, the culture medium was removed, and cells were incubated with DCFH-DA (10 μM) diluted in serum-free medium at 37 °C for 30 min in the dark. Cells were subsequently washed with PBS three times. Fluorescence images were acquired using an inverted fluorescence microscope (green channel). For flow cytometry quantification, cells were collected and the fluorescence intensity was analyzed using the FITC channel.

### 2.8 MTT cell viability assay

4T1 cells were seeded in 96-well plates at 1 × 10^4^ cells per well and allowed to adhere. Cells were then treated with RKLAHE–Mn at various concentrations (25, 50, 100, 150, and 200 μM; corresponding Mn concentrations 23, 46, 92, 138, and 184 μM, respectively) for 24 h. MTT working solution was added and incubated for 4 h. The supernatant was removed, and the formazan crystals were dissolved in DMSO. Absorbance was measured at 490 nm using a microplate reader. Cell viability was calculated as the percentage of the untreated control.

### 2.9 Cellular NADPH and ATP quantification

#### 2.9.1 NADPH assay

4T1 cells were seeded in 6-well plates at 1 × 10^6^ cells per well and cultured overnight. Cells were treated with RKLAHE–Mn (100 μM; corresponding Mn concentration 92 μM) for 12 h. NADPH levels were determined using a commercial NADP^+^/NADPH assay kit following the manufacturer's instructions. Briefly, cells were lysed to obtain extracts and deproteinized prior to measurement. Samples and working reagents were added into 96-well plates, and absorbance was recorded at 450 nm. NADPH concentrations were calculated based on the standard curve.

##### 2.9.1.1 ATP assay

For intracellular ATP determination, 4T1 cells were seeded in black opaque 96-well plates at 5 × 10^5^ cells per well. After treatment, intracellular ATP was quantified using an ATP assay kit according to the manufacturer's instructions. Cells were lysed using ATP lysis buffer, and ATP levels were measured based on the corresponding luminescence mode specified by the kit. For extracellular ATP measurement, culture supernatants were collected after treatment and analyzed following the kit protocol.

### 2.10 Mitochondria/lysosome staining and colocalization analysis

4T1 cells were seeded in confocal dishes at 1 × 10^5^ cells per dish. After incubation with RKLAHE^NBD^ (50 μM), cells were stained with MitoTracker (100 nM) at 37 °C for 30 min, washed with PBS, and counterstained with Hoechst 33342. For lysosome staining, cells were treated under the same conditions and stained with LysoTracker for 30 min, followed by nuclear staining. Live-cell confocal images were acquired immediately after staining. Colocalization was quantified by calculating the Pearson's correlation coefficient using image analysis software.

### 2.11 Apoptosis analysis

4T1 cells were seeded in 12-well plates at 1 × 10^5^ cells per well. After adherence, cells were treated with RKLAHE–Mn (100 μM; Mn 92 μM) for 24 h. Cells were collected by trypsinization, washed with PBS, and stained using an Annexin V-FITC/PI apoptosis detection kit according to the manufacturer's protocol. Apoptotic populations were quantified by flow cytometry.

### 2.12 Western blotting

4T1 cells were seeded in 6-well plates at 1 × 10^6^ cells per well and treated with RKLAHE–Mn (100 μM; Mn 92 μM) for 12 h. Cells were harvested and lysed in an appropriate lysis buffer. Protein samples were separated by SDS–PAGE and transferred onto PVDF membranes. Membranes were blocked with 5% non-fat milk for 1 h and incubated with the indicated primary antibodies. GAPDH was used as the loading control. After incubation with HRP-conjugated secondary antibodies for 1 h, protein bands were visualized using an imaging system.

### 2.13 Cytosolic mtDNA quantification and qPCR

4T1 cells were seeded in 6-well plates at 1 × 10^6^ cells per well and treated with RKLAHE–Mn (100 μM; corresponding Mn concentration 92 μM) for 8 h. Cells were detached with trypsin and neutralized with an equal volume of complete medium. The cell suspension was collected and split equally into two 1.5 mL microcentrifuge tubes: Tube 1 for total DNA preparation and Tube 2 for cytosolic DNA preparation. Cells were pelleted at 200 × g for 5 min, and the supernatant was discarded.

To obtain the cytosolic fraction, the cell pellet in Tube 2 was gently resuspended in 250 μL Buffer A and incubated at room temperature for 10 min to selectively permeabilize the plasma membrane (Buffer A: 150 mM NaCl, 50 mM HEPES, 25 μg mL^−1^ digitonin, pH 7.4). The lysate was centrifuged at 1000 × g for 10 min to remove intact cells and nuclei. The supernatant was transferred to a new tube and further centrifuged at 15,000 × g for 10 min to remove residual debris and organelles, and the final clarified supernatant was collected as the cytosolic fraction.

DNA from the cytosolic fraction (Tube 2) and total cellular DNA (Tube 1) were purified using a column-based DNA extraction kit according to the manufacturer's instructions. Purified DNA was quantified by a NanoDrop spectrophotometer, and cytosolic DNA concentration (ng μL^−1^) was recorded.

Cytosolic mtDNA was quantified by real-time quantitative PCR (qPCR). The mitochondrial gene ND1 was amplified using DNA purified from the cytosolic fraction as the template, while the nuclear gene 18S was amplified from total cellular DNA for normalization. qPCR was performed using a SYBR Green master mix, and relative cytosolic mtDNA levels were calculated using the 2^−^ΔΔCt method.

**Table:** 

Gene	Primers
ND1 Forward Primers	CCCTAAAACCCGCCACATCT
ND1Reverse Primers	GAGCGATGGTGAGAGCTAAGGT
18S rRNA Forward Primers	CAGCCACCCGAGATTGAGCA
18S rRNA Reverse Primers	TAGTAGCGACGGGCGGTGT

### 2.14 Immunofluorescence staining for CRT exposure and HMGB1 translocation

4T1 cells were seeded in confocal dishes at 1 × 10^5^ cells per dish and cultured overnight. Cells were treated with RKLAHE–Mn (100 μM; Mn 92 μM) for 8 h, fixed with paraformaldehyde, and washed with PBS. After blocking with 5% BSA for 30 min, cells were incubated with an anti-CRT primary antibody at 4 °C overnight, followed by incubation with the corresponding fluorescent secondary antibody for 1 h. Nuclei were counterstained with DAPI, and images were acquired using a confocal microscope.

HMGB1 staining was performed similarly, except that cells were permeabilized with 0.1% Triton X-100 for 5 min after fixation. Cells were then incubated with an anti-HMGB1 primary antibody at 4 °C overnight, followed by fluorescent secondary antibody incubation for 1 h. Nuclei were counterstained with DAPI, and images were acquired using an inverted fluorescence microscope.

### 2.15 DC2.4 maturation and cytokine measurement

DC2.4 cells were seeded in 12-well plates at 1 × 10^5^ cells per well and treated with RKLAHE–Mn (50 μM; Mn 46 μM) for 24 h. Cells were harvested and stained with fluorophore-conjugated antibodies against maturation markers (CD80, CD86, and MHC II) for 20 min at room temperature in the dark. After washing, samples were analyzed by flow cytometry.

For cytokine analysis, cell culture supernatants were collected after treatment and the levels of TNF-α, IL-6, and CXCL10 were quantified by ELISA according to the manufacturers’ protocols. Cytokine concentrations were calculated from standard curves.

### 2.16 Antigen cross-presentation assay

DC2.4 cells were seeded in 12-well plates at 1 × 10^5^ cells per well. Cells were incubated with OVA_257–264_ peptide (5 μg mL^−1^) and simultaneously treated with RKLAHE–Mn (50 μM; Mn 46 μM) for 24 h. Cells were then harvested and stained for surface expression of the H-2K^b^–SIINFEKL complex using flow cytometry following standard staining procedures.

### 2.17 Ex vivo fluorescence biodistribution imaging

When 4T1 tumors reached approximately 150–200 mm^3^, mice were randomized into groups and intravenously injected with IR820, RKLAHE^IR820^, or RKLAHE–Mn^IR820^ (IR820 concentration: 0.8 mg mL^−1^). At 24 h post-injection, major organs and tumors were excised for ex vivo fluorescence imaging. Fluorescence signals were quantified by region-of-interest (ROI) analysis using the imaging software.

### 2.18 4T1 tumor model and *in vivo* immune analysis

Female BALB/c mice (6–8 weeks old) were inoculated subcutaneously with 4T1 cells (1 × 10^6^ cells in 100 μL PBS). When tumors reached approximately 50 mm^3^, mice were randomized into groups and treated as indicated. Tumor length (L) and width (W) were measured every 4 days, and tumor volume was calculated as V = (L × W^2^)/2; body weight was monitored throughout the study. For dose normalization, RKLAHE–Mn was administered at 5 mg Mn kg^−1^, corresponding to 16.7 mg peptide kg^−1^, and the RKLAHE and MnCl_2_·4H_2_O control groups were dosed to match the peptide-equivalent (16.7 mg kg^−1^) or Mn-equivalent (5 mg kg^−1^), respectively; anti–PD-L1 was administered intraperitoneally at 100 μg per mouse (100 μL of 1 mg mL^−1^).

For immune profiling, spleens were mechanically dissociated followed by red blood cell lysis to obtain single-cell suspensions and stained with anti-CD11c, anti-CD80, and anti-CD86. Tumor-draining lymph nodes were processed through a cell strainer to prepare single-cell suspensions and stained with anti-CD3 and anti-CD8. Tumor tissues were minced and enzymatically digested into single-cell suspensions, followed by surface staining with anti-CD45, anti-CD3, anti-CD4, anti-CD8, anti-CD11b, anti-F4/80, anti-CD11c, anti-MHC II, anti-CD80, anti-CD86, and anti-NKp46 (as indicated) for flow cytometric analysis of immune cell infiltration and activation.

### 2.19 Statistical analysis

All data are presented as mean ± SEM, unless otherwise indicated. Statistical analyses were performed using GraphPad Prism 8.0 or Microsoft Excel 2016. Comparisons among multiple groups were performed using one-way ANOVA. Statistical significance was defined as: ∗P < 0.05, ∗∗P < 0.01, ∗∗∗P < 0.001, and ∗∗∗∗P < 0.0001.

## 3 Results and discussion

### 3.1 Construction and characterization of Mn^2+^-coordinated peptide nanoassemblies

**Fig. 1:**
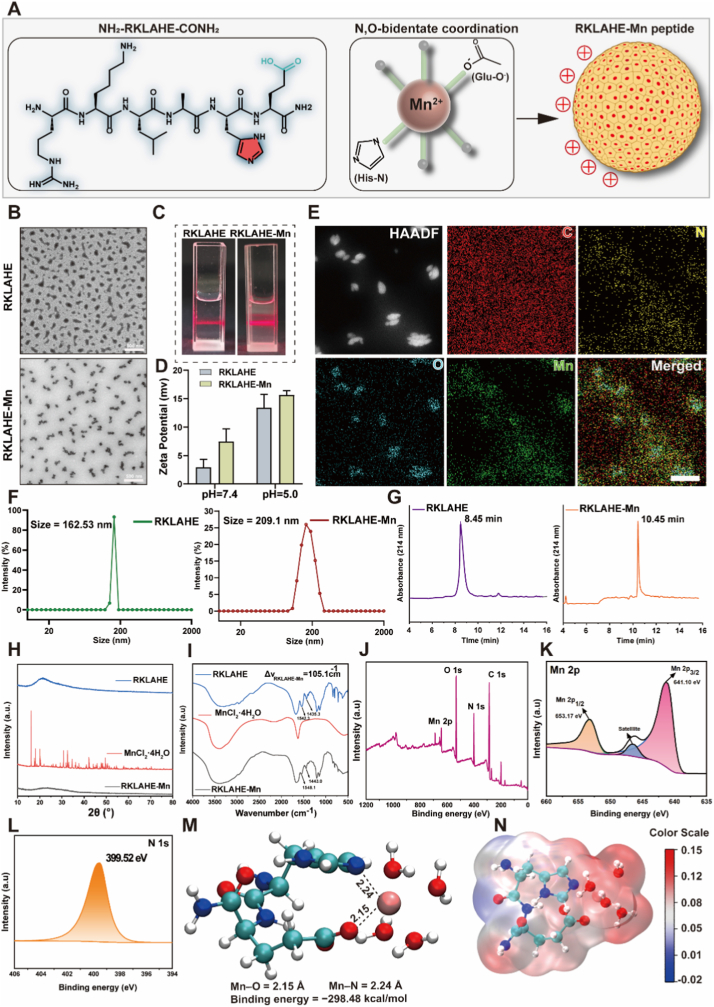
**Construction and characterization of Mn^2+^-crosslinked RKLAHE nanoassemblies**. (A) Design concept of Mn^2+^-mediated coordination crosslinking via peptide side-chain N/O donors (imidazole N from His and carboxylate O from Glu), forming N,O-bidentate coordination. (B) Representative TEM images of RKLAHE and RKLAHE–Mn. (C) Photographs of the Tyndall effect for RKLAHE and RKLAHE–Mn dispersions. (D) Zeta potentials of RKLAHE and RKLAHE–Mn measured at different pH values. (E) HAADF-STEM image of RKLAHE–Mn and corresponding EDS elemental mappings of C, N, O, and Mn (scale bar = 300 nm). (F) Hydrodynamic size distributions of RKLAHE and RKLAHE–Mn in water. (G) Analytical HPLC chromatograms of RKLAHE and RKLAHE–Mn monitored at 214 nm. (H) XRD patterns of RKLAHE, MnCl_2_·4H_2_O, and RKLAHE–Mn. (I) FT-IR spectra of RKLAHE, MnCl_2_·4H_2_O, and RKLAHE–Mn. (J) XPS survey spectrum of RKLAHE–Mn. (K–L) High-resolution XPS spectra of RKLAHE–Mn: Mn 2p (K), N 1s (L). (M) DFT-optimized Mn–His/Glu coordination model. Mn–N = 2.24 Å, Mn–O = 2.15 Å, and ΔE_bind = −298.48 kcal mol^−1^. (N) Electrostatic potential surface of the optimized model.

The programmable chemical addressability of natural amino acid side chains offers a versatile platform for constructing metal–organic hybrid nanostructures [32]. Guided by this principle, we designed a hexapeptide, NH_2_–RKLAHE–CONH_2_, engineered to leverage the imidazole nitrogen of Histidine (His) and the carboxylate oxygen of Glutamic acid (Glu) as canonical N/O donor sites. We hypothesized that Mn^2+^ would drive supramolecular self-assembly via N,O-bidentate coordination, thereby promoting the association of the peptide into stable, functional nanoassemblies (Fig. 1A). The identity of the synthesized peptide was first verified by mass spectrometry (Fig. S1). To optimize the formulation for subsequent biological studies and assess the contribution of the E/H residues to Mn coordination, we compared Mn loading efficiencies of RKLAHE-Mn and RKLAAA-Mn at different peptide/Mn^2+^ feed molar ratios. RKLAHE consistently showed higher Mn loading than RKLAAA, supporting the involvement of the E/H residues in Mn coordination. Accordingly, the 1:4 peptide/Mn^2+^ ratio was selected for subsequent experiments. (Fig. S2).

We next examined the assembly behavior by TEM. The Mn-free RKLAHE predominantly formed dispersed particulate nanoaggregates, whereas Mn^2+^ coordination markedly remodeled the morphology into more uniform and smaller nanoparticles with a distinct chain-like appearance, suggesting that Mn^2+^ coordination promotes ordered interpeptide association and assembly reconstruction (Fig. 1B). Consistently, high-resolution TEM did not reveal continuous lattice fringes, and the corresponding electron diffraction pattern exhibited diffuse halo rings, indicating that RKLAHE–Mn is largely amorphous with low crystallinity (Fig. S3). Colloidal scattering characteristics further supported nanoassembly formation. Compared with RKLAHE, RKLAHE–Mn displayed a more pronounced Tyndall effect under laser irradiation, indicative of a stable dispersion of nanoscale assemblies (Fig. 1C). Given that metal coordination may alter surface electrostatics, we measured ζ-potential across different pH values. At pH 7.4, RKLAHE–Mn exhibited an overall higher ζ-potential than RKLAHE, implying a coordination-induced surface charge reconfiguration. Considering the sequence features (positively charged Arg/Lys versus negatively charged Glu), this shift may arise from Mn^2+^ coordination with Glu/His partially attenuating the contribution of carboxylate negative charges while facilitating exposure of cationic side chains at the assembly surface. Notably, at pH 5.0 both RKLAHE and RKLAHE–Mn became more positively charged, which is consistent with enhanced protonation of His under acidic conditions (Fig. 1D). To exclude the possibility that Mn^2+^ was merely adsorbed rather than structurally integrated, HAADF-STEM coupled with EDS mapping was performed. The spatial distributions of C, N, and O overlapped strongly with Mn, and Mn signals were uniformly distributed within nanoparticle regions (Fig. 1E). EDS spectral analysis further revealed clear Mn characteristic peaks in addition to peptide-derived elements, confirming the presence of Mn in the assemblies. Quantitatively, Mn accounted for ∼4.92 wt% of the sample, consistent with the observed Mn–peptide spatial colocalization and supporting stable incorporation of Mn^2+^ into RKLAHE nanoassemblies via coordination (Fig. S4).

Dynamic light scattering showed that RKLAHE formed nanoscale aggregates in water with an average hydrodynamic diameter of ∼162.5 nm. Upon Mn^2+^ incorporation, the hydrodynamic diameter slightly increased to ∼209.1 nm, potentially reflecting the Mn^2+^-associated hydration shell and the formation of more ordered bead-chain assemblies that increase the apparent hydrodynamic size (Fig. 1F). Analytical HPLC monitored at 214 nm revealed distinct retention times for RKLAHE (8.45 min) and RKLAHE–Mn (10.45 min), with Mn^2+^ coordination leading to an increased retention time, suggesting an overall change in hydrophobicity after coordination-driven assembly (Fig. 1G). We next examined the stability and release behavior of the metallopeptide assemblies. In serum-containing medium, both RKLAHE and RKLAHE–Mn maintained relatively stable hydrodynamic sizes over 7 days, supporting good colloidal stability under biologically relevant conditions (Fig. S5). We then evaluated Mn release under different pH conditions and found that RKLAHE–Mn exhibited a clear acid-responsive release profile, with substantially greater cumulative Mn release at pH 5.0 than at pH 7.4 over 24 h. By contrast, HPLC traces recorded after 24 h incubation at pH 5.0 or 7.4 still retained a dominant peptide peak, with no obvious degradation-related fragmentation pattern (Fig. S5). These results suggest that Mn coordination preserves the structural stability of the peptide assembly while allowing preferential Mn release under acidic conditions. To probe whether Mn^2+^ coordination altered peptide secondary structure, far-UV circular dichroism (CD) spectra were collected. As shown in Fig. S5, RKLAHE and RKLAHE–Mn exhibited highly similar spectral profiles, featuring a pronounced negative band around 198–200 nm and a positive contribution near ∼220 nm, consistent with predominantly random-coil conformations accompanied by partial ordered structural contributions. Importantly, Mn^2+^ coordination caused only minor changes in peak intensity and position, indicating that Mn^2+^ does not induce major secondary-structure rearrangement of the peptide backbone but may instead introduce local conformational adjustments or stabilize specific conformational states.

XRD analysis further supported the noncrystalline nature of RKLAHE–Mn. In contrast to MnCl_2_·4H_2_O, RKLAHE–Mn did not display sharp diffraction peaks but rather a broad diffuse pattern, indicative of an amorphous structure (Fig. 1H). FT-IR spectroscopy was then used to investigate coordination sites and binding modes. For RKLAHE, characteristic carboxylate –COO^-^ asymmetric (*ν*_as) and symmetric (*ν*_s) stretching bands were observed at 1542.3 cm^−1^ and 1435.3 cm^−1^, respectively. After Mn^2+^ incorporation, these bands shifted to 1548.1 cm^−1^ and 1443.0 cm^−1^, suggesting a coordination-induced change in the local electronic environment of the carboxylate group and supporting the participation of Glu side-chain carboxylate in Mn^2+^ binding (Fig. 1I). The calculated Δν (*ν*_as − *ν*_s) of 105.1 cm^−1^ falls within a low-Δν regime commonly associated with bidentate chelating carboxylate–metal coordination, consistent with an N,O-bidentate coordination model. Moreover, XPS further corroborated successful Mn incorporation. The survey spectrum revealed signals of C, N, O, and Mn, confirming Mn integration into RKLAHE–Mn (Fig. 1J). In the high-resolution Mn 2p spectrum, characteristic peaks corresponding to Mn 2p_3_/_2_ and Mn 2p_1_/_2_ (approximately 641.10 eV and 653.17 eV), together with satellite features, were consistent with Mn(II), suggesting that Mn predominantly remains in the Mn^2+^ state within the assemblies (Fig. 1K). Meanwhile, N 1s (∼399.52 eV), O 1s (∼531.17 eV), and C 1s components (including O=C–N) reflected the peptide backbone and oxygen-/nitrogen-containing functional groups (Fig. 1L and Fig. S7). To further elucidate the local coordination mode between Mn(II) and the peptide, DFT calculations were performed. The optimized structure showed that Mn(II) could be simultaneously coordinated by the imidazole N atom of His and the carboxylate O atom of Glu, thereby forming a stable local N,O-coordination environment. In the optimized model, the Mn–N and Mn–O bond lengths were calculated to be 2.24 Å and 2.15 Å, respectively, which fall within a reasonable range for metal coordination and support the cooperative involvement of His and Glu in Mn binding (Fig. 1M). In addition, the calculated binding energy was −298.48 kcal mol^−1^, indicating a thermodynamically favorable coordination interaction. Furthermore, the electrostatic potential surface analysis revealed an electronically favorable environment around the coordinating atoms and the Mn center, further supporting the rationality of the proposed local coordination configuration (Fig. 1N). Together with FT-IR peak shifts and Δν analysis, these data collectively support coordination interactions between Mn^2+^ and peptide N/O donor sites. Collectively, these data confirm the successful construction of RKLAHE–Mn nanoassemblies driven by specific N,O-bidentate coordination.

### 3.2 RKLAHE–Mn nanoassemblies enhance cellular uptake and induce oxidative stress

**Fig. 2:**
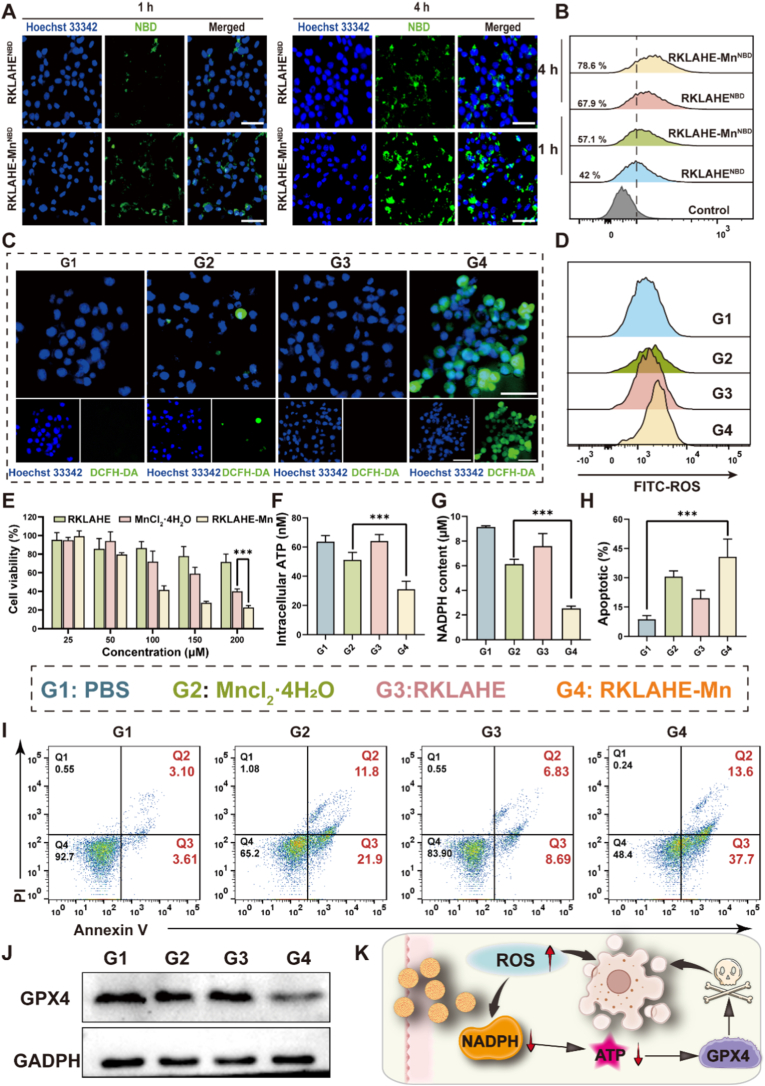
**RKLAHE–Mn enhances cellular uptake and induces redox imbalance to promote tumor cell apoptosis.** (A) Representative fluorescence images of 4T1 cells after co-incubation with RKLAHE^NBD^ or RKLAHE–Mn^NBD^ for 1 h and 4 h; nuclei were stained with Hoechst 33342 (scale bar = 25 μm). (B) Flow cytometry analysis quantifying intracellular uptake of RKLAHE^NBD^ and RKLAHE–Mn^NBD^ in 4T1 cells at 1 h and 4 h. (C) Fluorescence imaging of intracellular reactive oxygen species (ROS) generation in 4T1 cells under different treatments (scale bar = 25 μm). (D) Flow cytometry quantification of ROS fluorescence intensity corresponding to (C). (E) Cell viability of 4T1 cells after 24 h treatment determined by the MTT assay. (F) Intracellular ATP levels in 4T1 cells after 12 h treatment. (G) Intracellular NADPH levels in 4T1 cells after 12 h treatment. (H–I) Quantification and representative flow cytometry plots of apoptosis in 4T1 cells after the indicated treatments. (J) GPX4 expression in 4T1 cells after indicated treatments. (K) Schematic illustration: enhanced uptake of RKLAHE–Mn leads to ROS elevation, NADPH/ATP depletion and GPX4 downregulation, collectively driving oxidative injury and cell death. Data are presented as mean ± SEM (n = 3).

To elucidate the impact of metal-coordination on cellular interactions, we synthesized NBD-labeled variants of the peptide (Fig. S8). Confocal microscopy and flow cytometry revealed a stark contrast in internalization efficiency: the coordinated RKLAHE-Mn nanoassemblies exhibited significantly higher accumulation in 4T1 cells compared to the free RKLAHE peptide (Fig. 2A and B). This enhanced uptake is attributed to the rigid supramolecular architecture and increased positive surface charge of the assembly, which likely facilitate improved interaction with the anionic cell membrane and subsequent endocytosis. Once internalized, the assembly functions as a potent generator of oxidative stress. Using the DCFH-DA probe, we observed that RKLAHE-Mn triggered a surge in intracellular reactive oxygen species (ROS) far exceeding that of free RKLAHE or MnCl_2_ controls (Fig. 2C and D). These findings support a synergistic mechanism in which the peptide scaffold not only delivers manganese into cells but also enhances its intracellular retention and localization, thereby amplifying ROS generation.

MTT assays demonstrated that RKLAHE-Mn induced a pronounced, dose-dependent reduction in cell viability, whereas free RKLAHE or MnCl_2_ alone had minimal effects and remained largely biocompatible. These results indicate that the observed cytotoxicity arises predominantly from the supramolecular assembly rather than from either individual component (Fig. 2E and Fig. S9). To assess biosafety toward normal cells, HUVEC viability was measured after treatment with RKLAHE, MnCl_2_·4H_2_O, and RKLAHE–Mn. RKLAHE alone showed low cytotoxicity across the tested concentrations, whereas free MnCl_2_·4H_2_O caused a concentration-dependent decrease in cell viability. In comparison, RKLAHE–Mn displayed reduced toxicity relative to free MnCl_2_·4H_2_O, supporting that peptide coordination improves the biocompatibility of the Mn-containing formulation (**Figure S10**). We further examined the cell-death–associated changes and observed a marked bioenergetic and redox imbalance. The ROS surge was accompanied by a rapid depletion of intracellular ATP and NADPH, two key cofactors for cellular repair processes and antioxidant maintenance (Fig. 2F and G). Consistently, we detected impairment of the GPX4 axis, a critical checkpoint limiting lipid peroxidation; Western blot analysis confirmed a significant reduction in GPX4 protein levels. (Fig. 2J). In parallel, Annexin V-FITC/PI flow cytometry revealed an increased fraction of Annexin V–positive cells, confirming enhanced apoptosis under RKLAHE-Mn treatment (Fig. 2H and I). Collectively, these results suggest that RKLAHE-Mn–induced cytotoxicity is driven by a cascade of oxidative stress, energy depletion, and compromised antioxidant defenses (Fig. 2K).

### 3.3 RKLAHE–Mn induces mitochondrial stress and activates mtDNA-mediated cGAS–STING signaling to drive ICD

**Fig. 3:**
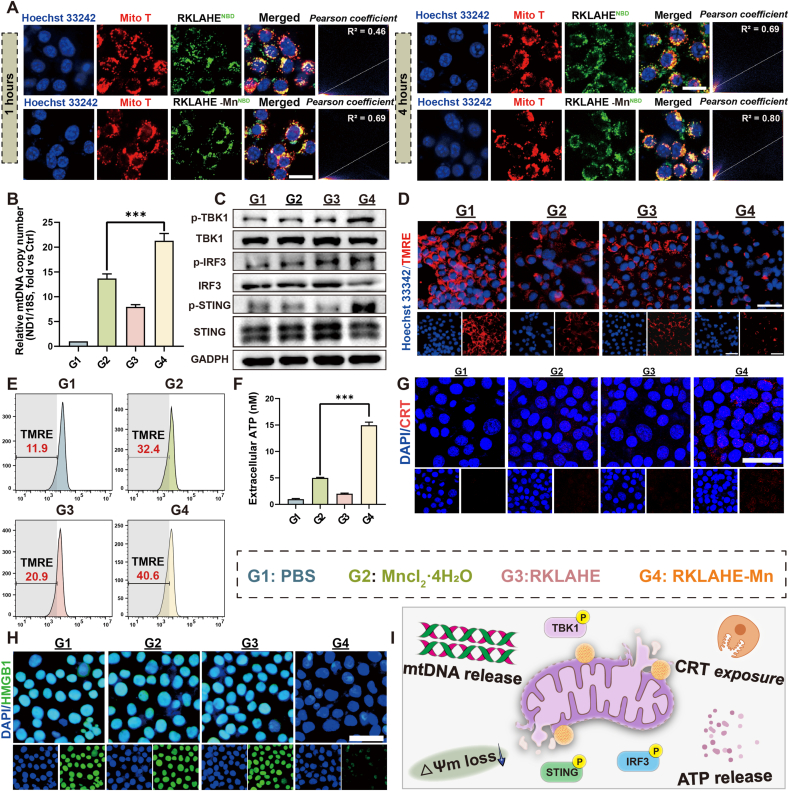
**RKLAHE–Mn induces mitochondrial stress and mtDNA release to activate the cGAS–STING axis, accompanied by immunogenic cell death associated signals**. (A) Confocal images of mitochondrial colocalization of RKLAHE^NBD^ or RKLAHE–Mn^NBD^ in 4T1 cells after 1 h and 4 h incubation. Mitochondria were stained with MitoTracker and nuclei were counterstained with Hoechst 33342; Pearson's correlation coefficients (right) were calculated to quantify colocalization (scale bar = 25 μm). (B) qPCR quantification of cytosolic mtDNA release. Cytosolic fractions were isolated after 12 h treatment, and DNA was extracted for qPCR analysis of mtDNA (ND1) with normalization to 18S. (C) Western blot analysis of STING pathway-related proteins after 12 h treatment. (D) Mitochondrial membrane potential assessment by TMRE staining after 12 h treatment with representative fluorescence images (scale bar = 25 μm). (E) Flow cytometry quantification of TMRE fluorescence intensity corresponding to (D). (F) Extracellular ATP levels in culture supernatants of 4T1 cells after the indicated treatments. (G) Confocal image of CRT to evaluate surface exposure of CRT after treatment (scale bar = 50 μm). (H) fluorescence imaging of HMGB1 to assess its intracellular localization changes after treatment (scale bar = 25 μm). (I) Proposed mechanism: RKLAHE–Mn induces mitochondrial stress and ΔΨm loss, promotes cytosolic mtDNA release and activates the cGAS–STING–TBK1–IRF3 axis, while eliciting ICD-associated signals, thereby facilitating immune activation. Data are presented as mean ± SEM (n = 3).

To delineate the intracellular trafficking route after cellular entry, we first performed colocalization analysis with lysosomes stained by LysoTracker. Confocal imaging revealed that RKLAHE^NBD^ exhibited substantial lysosomal colocalization at 1 h (Pearson correlation coefficient, R = 0.57) and remained appreciable at 4 h (R = 0.50), suggesting predominant uptake via endocytosis followed by accumulation within the lysosomal compartment. In contrast, RKLAHE–Mn^NBD^ showed a lower degree of lysosomal colocalization at 1 h (R = 0.40), which further decreased at 4 h (R = 0.23), accompanied by a more dispersed cytosolic fluorescence distribution, indicative of enhanced lysosomal escape after Mn^2+^ coordination-driven assembly (Fig. S11). This behavior may be associated with the presence of His residues in RKLAHE, whose imidazole side chains can undergo protonation in the acidic lysosomal milieu, thereby increasing buffering capacity and surface positive charge; such pH responsiveness is known to facilitate endosomal membrane perturbation and reduce lysosomal retention. Motivated by the improved lysosomal escape, we next examined mitochondrial association using MitoTracker Red staining. RKLAHE^NBD^ displayed a moderate level of mitochondrial colocalization, whereas RKLAHE–Mn^NBD^ showed markedly stronger overlap with mitochondria. Quantitative analysis yielded a Pearson coefficient of R = 0.46 for RKLAHE^NBD^, which increased to R = 0.69–0.80 for RKLAHE–Mn^NBD^ from 1 h to 4 h (Fig. 3A). To specifically address whether mitochondrial association was mediated by electrostatic attraction, we generated a charge-attenuated control peptide, QQLAHE^NBD^, by replacing the N-terminal Lys/Arg residues with neutral Gln residues. QQLAHE^NBD^ showed only minimal mitochondrial colocalization at both 1 h and 4 h (Pearson's R = 0.11 and 0.18, respectively), which was markedly lower than that of RKLAHE^NBD^ and RKLAHE–Mn^NBD^ (Fig. S12). These results support that the mitochondrial association of the RKLAHE-based system is predominantly driven by electrostatic interaction arising from its cationic residues. To further verify that the enhanced mitochondrial colocalization translated into actual mitochondrial accumulation, we isolated mitochondrial fractions and quantified Mn content by ICP-MS. As shown in Fig. S13, cells treated with RKLAHE–Mn exhibited markedly higher mitochondrial Mn levels than those treated with free MnCl_2_·4H_2_O, whereas only negligible background signals were detected in the Control and RKLAHE groups. These data provide direct quantitative evidence that Mn coordination-driven assembly promotes mitochondrial Mn accumulation, further supporting that the mitochondrial association of the RKLAHE-based system is mediated by electrostatically favored interactions.

Mitochondrial depolarization is frequently accompanied by altered membrane permeability and mtDNA release. We therefore quantified cytosolic mtDNA levels and found that RKLAHE–Mn significantly increased the relative cytosolic abundance of mtDNA (ND1) compared with controls, indicating mtDNA accumulation in the cytosol (Fig. 3B). To validate downstream signal transduction, we examined phosphorylation of key proteins within the STING pathway. Western blotting showed pronounced upregulation of p-STING, p-TBK1, and p-IRF3 upon RKLAHE–Mn treatment, which was further supported by densitometric analysis of the corresponding phosphorylation ratios (Fig. 3C and Fig. S14) To further determine whether this pathway activation was specific to Mn rather than a general consequence of metal coordination, we introduced Fe-coordinated and other metal-treated controls and compared their effects on STING signaling. Among the tested formulations, RKLAHE–Mn produced the strongest activation of the pathway, as evidenced by the marked upregulation of p-STING and p-TBK1, whereas Fe-coordinated or other metal controls showed little or no comparable activation (Fig. S15). These results support that the STING-activating effect of the metallopeptide system is predominantly Mn-specific. Given that STING activation has been reported to induce PD-L1 upregulation, we next examined PD-L1 expression after treatment with the indicated formulations. Notably, PD-L1 was markedly increased in the RKLAHE–Mn group, whereas RKLAHE alone and MnCl_2_·4H_2_O produced no comparable change relative to the control (Fig. S16). This result further supports that Mn-specific activation of STING signaling is accompanied by PD-L1 upregulation, thereby providing a rationale for the subsequent combination with αPD-L1 therapy *in vivo*. Together, these data support that RKLAHE–Mn induces mitochondrial damage, promotes mtDNA leakage, and consequently triggers innate immune signaling. Given the mitochondrial enrichment profile, mitochondrial membrane potential (ΔΨm) was further assessed using TMRE staining. Fluorescence imaging revealed an evident reduction of TMRE signal in RKLAHE–Mn-treated cells relative to PBS, which was corroborated by flow cytometric quantification showing a significant decrease in TMRE intensity, RKLAHE–Mn elicited the most pronounced ΔΨm dissipation among the tested groups (Fig. 3D–E and Fig. S17). These findings demonstrate that Mn^2+^-coordinated assemblies induce robust mitochondrial depolarization and mitochondrial dysfunction.

We next evaluated canonical ICD-associated danger signals. RKLAHE–Mn markedly increased extracellular ATP levels, indicating enhanced release of stress-associated chemoattractant cues (Fig. 3F). In parallel, immunofluorescence staining showed a substantial increase of CRT signal on the cell surface in the RKLAHE–Mn group, consistent with typical “eat-me” signal exposure (Fig. 3G). To further address the temporal persistence of CRT exposure, we quantified surface CRT by flow cytometry after 24 h treatment. Consistent with the 8 h immunofluorescence results, RKLAHE–Mn produced the highest proportion of CRT-positive cells at 24 h, whereas the other groups showed only modest increases relative to the control (Fig. S18). Furthermore, HMGB1 immunostaining revealed that HMGB1 was largely confined to the nucleus in control cells, displaying strong overlap with DAPI, whereas RKLAHE–Mn treatment resulted in weakened nuclear HMGB1 staining, suggesting HMGB1 release from the nucleus (Fig. 3H). Collectively, these results integrate mitochondrial depolarization, cytosolic mtDNA accumulation, and STING activation, indicating that RKLAHE–Mn engages mtDNA-mediated activation of the cGAS–STING pathway while concurrently eliciting ICD associated stress programs (Fig. 3I).

### 3.4 RKLAHE–Mn orchestrates DC maturation and antigen cross-presentation via cGAS–STING activation

**Fig. 4:**
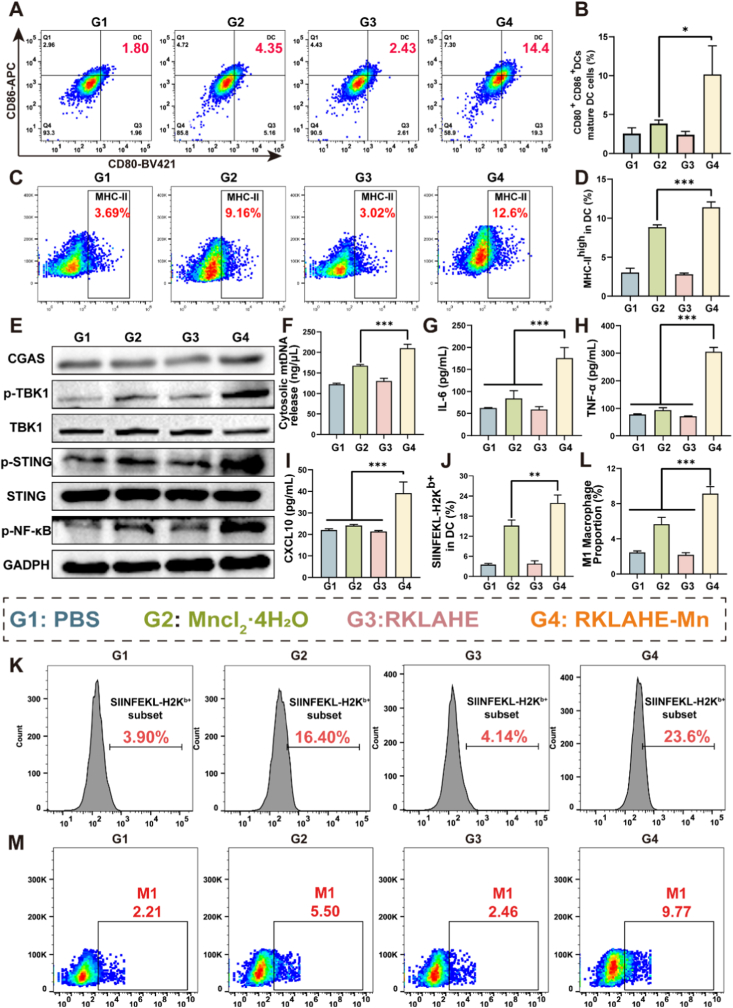
**RKLAHE–Mn promotes dendritic cell activation and enhances cross-presentation, accompanied by cGAS–STING activation and upregulated inflammatory cytokines.** (A) Flow cytometry analysis of the percentage of CD80^+^CD86^+^ DC2.4 cells after 24 h under the indicated treatments. (B) Quantification of CD80^+^CD86^+^ DC2.4 cells corresponding to (A). (C) Flow cytometry analysis of MHC II expression on DC2.4 cells after 24 h treatment. (D) Quantification of MHC II^+^ DC2.4 cells corresponding to (C). (E) Western blot analysis of cGAS–STING pathway-related proteins in antigen-presenting cells after 12 h treatment. (F) qPCR quantification of cytosolic mtDNA levels in DC2.4 cells after 8 h treatment. (G–I) ELISA measurement of IL-6 (G), TNF-α (H), and CXCL10 (I) secreted in DC2.4 culture supernatants after 24 h treatment. (J) Quantification of H-2K^b^–SIINFEKL (OVA_257–264_)-positive DC2.4 cells after 24 h treatment, indicating MHC I antigen cross-presentation capacity. (L) Quantification of CD86^+^ RAW264.7 cells after 24 h treatment. (K). Representative flow cytometry histograms showing the H-2K^b^–SIINFEKL-positive DC2.4 population after 24 h treatment (M) Representative flow cytometry plots showing CD86 expression in RAW264.7 cells after 24 h treatment. Data are presented as mean ± SEM (n = 3).

Effective cancer immunotherapy relies on the ability of adjuvants to stimulate professional antigen-presenting cells (APCs) without compromising their viability. We first confirmed that RKLAHE–Mn exhibits excellent cytocompatibility with DC2.4 cells across a broad concentration range (25–75 μM), ensuring a safe therapeutic window (Fig. S19). Crucially, the assembly acted as a potent maturation stimulus. Flow cytometry demonstrated that RKLAHE-Mn treatment significantly expanded the population of DCs co-expressing costimulatory markers CD80 and CD86 (rising from ∼1.80% to ∼14.4%) and upregulated surface MHC II (Fig. 4A–D). This synchronized expression of critical costimulatory molecules and antigen-presentation machinery suggests that RKLAHE–Mn effectively licenses DCs for T-cell priming. Mechanistically, we attributed this immunogenic phenotype to the activation of the cGAS-STING cytosolic surveillance pathway. RKLAHE-Mn treatment induced a significant accumulation of cytosolic mtDNA, which served as a danger signal to trigger the phosphorylation of STING pathway components (Fig. 4E and F). This innate sensing translated into a robust proinflammatory secretome, characterized by the elevated release of IL-6, TNF-α, and the T-cell chemoattractant CXCL10 (Fig. 4G–I).

Cross-presentation is a key prerequisite for priming CD8^+^ T-cell responses. Using the canonical OVA_257–264_ (SIINFEKL) epitope as a model antigen, we observed that RKLAHE–Mn markedly increased the abundance of surface H-2K^b^–SIINFEKL complexes relative to controls (Fig. 4J and K). These data suggest enhanced cytosolic antigen routing and proteasome-dependent processing, thereby facilitating MHC-I loading. Consistently, the immunostimulatory activity extended to the innate compartment, as RAW264.7 macrophages exhibited a shift toward an activated CD86^+^ phenotype (Fig. 4L and M). Collectively, these findings identify RKLAHE–Mn as a dual-function adjuvant that promotes immune cell maturation via the cGAS-STING pathway while concurrently strengthening the cross-presentation axis required for systemic antitumor immunity.

### 3.5 RKLAHE–Mn synergizes with anti–PD-L1 to enhance antitumor efficacy *in vivo*

**Fig. 5:**
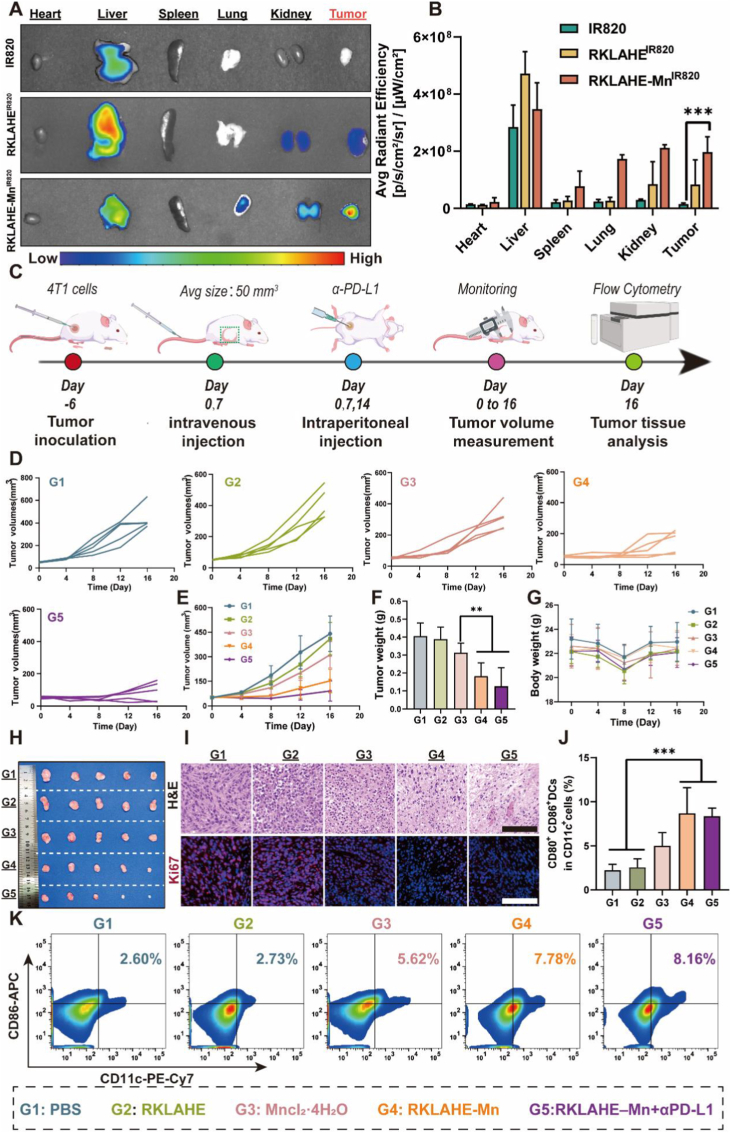
RKLAHE–Mn synergizes with anti–PD-L1 to enhance antitumor efficacy in 4T1 tumor-bearing mice. (A) Ex vivo fluorescence imaging of major organs and tumors at 24 h after intravenous injection of IR820, RKLAHE^IR820^, or RKLAHE–Mn^IR820^ in 4T1 tumor-bearing mice (n = 3). (B) semi-quantification of fluorescence signals in major organs and tumors corresponding to (A). (C) Schematic timeline of the *in vivo* treatment regimen for RKLAHE–Mn in combination with anti–PD-L1. (D) Individual tumor growth curves for each group (each line represents one mouse). (E) Mean tumor volume over time for each group. (F) Tumor weights collected at the endpoint. (G) Body weight changes during the treatment period. (H) Representative photographs of excised tumors after different treatments. (I) Representative H&E staining and Ki67 immunofluorescence images of tumor sections after the indicated treatments (scale bar = 100 μm). (J–K) Representative flow cytometry plots and quantification of CD80^+^CD86^+^ cells in CD11c^+^ dendritic cells in spleens after treatment. Data are presented as mean ± SEM (n = 5).

Efficient tumor accumulation of nanoformulations is a prerequisite for achieving robust therapeutic outcomes. To enable noninvasive tracking of *in vivo* biodistribution, we incorporated IR820, a near-infrared fluorescent tracer known for its deep-tissue imaging capability. *Ex vivo* fluorescence imaging revealed that free IR820 was predominantly distributed in metabolic organs such as the liver, with relatively weak signals in tumors. In contrast, both RKLAHE^IR820^ and RKLAHE–Mn^IR820^ exhibited markedly stronger fluorescence in tumor tissues. Semi-quantification further confirmed that RKLAHE–Mn^IR820^ achieved significantly higher tumor accumulation than free IR820 and overall outperformed RKLAHE^IR820^ (Fig. 5A and B). These results suggest that Mn^2+^-coordination-enhanced peptide assembly may improve structural stability and prolong systemic circulation, thereby facilitating tumor recruitment. Given that STING activation can be associated with adaptive immune resistance, including PD-L1 upregulation, we established a 4T1 tumor-bearing mouse model and evaluated RKLAHE–Mn in combination with anti–PD-L1 (Fig. 5C).Tumor growth curves showed rapid progression in the PBS control group, whereas RKLAHE–Mn treatment significantly delayed tumor growth; the combination with anti–PD-L1 further strengthened tumor suppression (Fig. 5D and E). Consistently, endpoint tumor weights were markedly reduced in the RKLAHE–Mn group and were further decreased in the combination group, indicating reliable *in vivo* antitumor efficacy and enhanced therapeutic benefit upon checkpoint blockade (Fig. 5F). Throughout the treatment period, body weights remained largely stable across groups, suggesting favorable systemic tolerability (Fig. 5G). Representative photographs of excised tumors also visually corroborated the reduced tumor burden in the RKLAHE–Mn and combination groups (Fig. 5H).

Histological analyses further supported these therapeutic effects. H&E staining revealed apparent alterations in cellular density and tissue architecture in tumors from RKLAHE–Mn-treated mice, which became more pronounced in the combination group. In parallel, Ki67 immunofluorescence signals were reduced relative to controls, indicating suppressed tumor cell proliferative activity and aligning with the observed tumor growth inhibition (Fig. 5I). Consistently, immunofluorescence staining of tumor sections revealed more evident CRT staining and enhanced cGAS and STING signals in the RKLAHE–Mn and RKLAHE–Mn+αPD-L1 groups relative to the PBS, RKLAHE, and MnCl_2_·4H_2_O groups, with the combination group showing the clearest overall tendency. In contrast, intracellular HMGB1 staining was reduced in the RKLAHE–Mn and RKLAHE–Mn+αPD-L1 groups, which is consistent with HMGB1 release during ICD (Fig. S20). In addition, spleen photographs and weight quantification showed enlarged spleens in PBS treated mice, whereas spleen weights tended to decrease with therapeutic intervention, with more evident reductions in the RKLAHE–Mn and combination groups (Fig. S21). This trend likely reflects alleviation of tumor burden-associated inflammation and myeloid expansion, consistent with the improved antitumor outcomes. Building on our *in vitro* evidence that RKLAHE–Mn promotes immune activation, we further examined dendritic cell maturation *in vivo*. Flow cytometry revealed a significant increase in the CD80^+^CD86^+^ mature subset among splenic CD11c^+^ dendritic cells after RKLAHE–Mn treatment, which was further augmented by combination with anti–PD-L1 (Fig. 5J and K). These data support that RKLAHE–Mn can promote APC activation *in vivo*, providing a mechanistic basis for its synergistic antitumor activity when combined with PD-L1 blockade.

### 3.6 RKLAHE–Mn enhances *in vivo* DC maturation and effector lymphocyte infiltration to amplify antitumor immunity

**Fig. 6:**
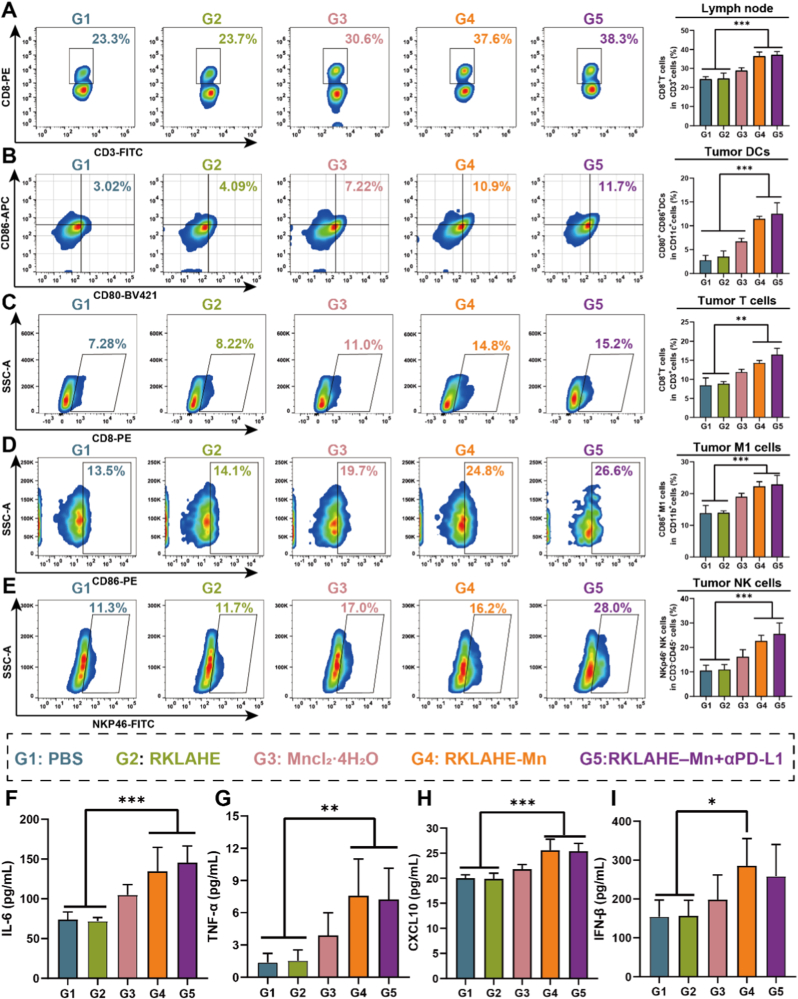
RKLAHE–Mn promotes *in vivo* DC maturation and enhances effector lymphocyte infiltration, thereby amplifying antitumor immune responses. (A) Representative flow cytometry plots and quantification of CD8^+^ T cells (CD8^+^ within CD3^+^) in tumor-draining lymph nodes. (B) Representative flow cytometry plots and quantification of CD80^+^CD86^+^ cells among CD11c^+^ dendritic cells in tumor tissues. (C) Representative flow cytometry plots and quantification of intratumoral CD8^+^ T cells (CD8^+^ within CD3^+^). (D) Representative flow cytometry plots and quantification of M1-like macrophages (CD86^+^ within CD11b^+^) in tumor tissues. (E) Representative flow cytometry plots and quantification of NK cells (NKp46^+^ within CD45^+^CD3^−^) in tumor tissues. (F–I) Serum levels of IL-6 (F), TNF-α (G), CXCL10 (H), and IFN-β (I) after the indicated treatments. Data are presented as mean ± SEM (n = 5).

To systematically assess RKLAHE–Mn–mediated immune remodeling, we performed flow cytometry at the treatment endpoint. Tumor-draining lymph nodes (dLNs) were analyzed to assess immune priming, followed by tumor tissues to quantify intratumoral immune infiltration and local immunosuppression. To systematically evaluate the remodeling effects of RKLAHE–Mn on the tumor immune microenvironment, we performed flow cytometric analysis of dLNs and tumor tissues at the treatment endpoint. In dLNs, the frequency of CD8^+^ T cells within the CD3^+^ T cell population was significantly increased in the RKLAHE–Mn group, rising from 23.3% in the PBS group to 37.6% with RKLAHE–Mn, and further to 38.3% in the combination group (Fig. 6A). These results suggest that RKLAHE–Mn enhances the initiation of tumor-specific immune responses, while PD-L1 blockade further amplifies this effect. Within tumors, RKLAHE–Mn markedly promoted dendritic cell activation. Compared with controls, the proportion of CD80^+^CD86^+^ mature subsets among intratumoral CD11c^+^ DCs was clearly elevated in the RKLAHE–Mn and combination groups (Fig. 6B and Fig. S22), indicating enhanced local DC maturation and co-stimulatory capacity that supports downstream T-cell–mediated antitumor immunity. Consistent with this DC maturation phenotype, intratumoral infiltration of effector T cells was also increased. The proportion of CD8^+^ T cells within CD3^+^ T cells rose from 7.28% in the PBS group to 14.8% in the RKLAHE–Mn group (Fig. 6C and Fig. S23), supporting that RKLAHE–Mn more effectively promotes recruitment and retention of effector CD8^+^ T cells in tumors.

We further assessed polarization changes in tumor-associated myeloid and innate immune compartments. Among CD11b^+^ myeloid cells, the fraction of CD86^+^ (M1-like) cells was significantly increased in the RKLAHE–Mn and combination groups, rising from 13.5% in PBS to 24.8% with RKLAHE–Mn and reaching 26.6% in the combination group (Fig. 6D and Fig. S24). This shift suggests a reprogramming of the tumor microenvironment from an immunosuppressive state toward a more proinflammatory and antitumor phenotype. In parallel, the abundance of intratumoral NKp46^+^ NK cells was significantly elevated in the combination group (Fig. 6E and Fig. S25), indicating that RKLAHE–Mn and anti–PD-L1 together further enhance innate effector engagement.

To corroborate systemic immune activation, we measured circulating cytokines and chemokines in serum. RKLAHE–Mn and the combination regimen significantly increased IL-6, TNF-α, CXCL10, and IFN-β levels (Fig. 6F–I), indicating activation of innate immune signaling and an amplified inflammatory program that may act upstream to drive DC maturation and effector T cell infiltration. Collectively, these data demonstrate that RKLAHE–Mn simultaneously enhances antigen presentation, promotes effector lymphocyte infiltration, and elevates proinflammatory cytokine profiles *in vivo*, thereby providing a mechanistic basis for its synergistic antitumor efficacy with PD-L1 blockade.

### 3.7 Biosafety evaluation

To assess the *in vivo* safety profile of the RKLAHE–Mn system, we examined hemocompatibility and potential toxicity to major organs. A red blood cell hemolysis assay was performed to evaluate membrane integrity upon exposure to different formulations. Compared with MnCl_2_·4H_2_O, both RKLAHE and RKLAHE–Mn caused negligible hemolysis across the tested concentration range, with overall hemolysis percentages remaining low; consistent with this, no apparent increase in red coloration of the supernatant was observed by visual inspection (Fig. S26). These results indicate favorable blood compatibility of the peptide-based assemblies.

To further evaluate potential systemic toxicity following repeated administration, major organs (heart, liver, spleen, lung, and kidney) were harvested at the end of treatment and subjected to H&E staining. Histological examination showed preserved tissue architecture across all groups, without obvious inflammatory cell infiltration, necrotic lesions, hemorrhage, or structural damage, and no marked differences were observed relative to the control group (Fig. S27). Collectively, the hemolysis and histopathological analyses support that RKLAHE–Mn exhibits good biosafety for *in vivo* application.

## 4 Conclusion

In summary, we have established an effective strategy for constructing metal-organic nanoassemblies by programming the coordination environment of Mn(II) with a designer hexapeptide. By exploiting the orthogonal binding affinities of histidine and glutamic acid, we demonstrated that dynamic N,O-bidentate chelation drives the self-assembly of RKLAHE–Mn into defined nanostructures with high metal loading and physiological stability. This approach effectively resolves the trade-off between structural durability and biodegradability, offering a scalable architecture validated by atomic-level spectroscopy and morphological analysis.

Biologically, RKLAHE–Mn functions as a potent, mitochondria-targeting immunomodulator. Upon internalization, the assembly triggers mitochondrial stress and cytosolic mtDNA leakage, which, combined with intracellular Mn^2+^ delivery, robustly activates the cGAS–STING pathway and induces immunogenic cell death. *In vivo*, this dual mechanism remodels the immunosuppressive tumor microenvironment by promoting dendritic cell maturation and CD8^+^ T cell infiltration. Consequently, RKLAHE–Mn elicits significant tumor regression, particularly when combined with anti–PD-L1 checkpoint blockade, while maintaining a favorable biosafety profile. Ultimately, this work positions coordination-driven peptide self-assembly as a versatile platform for metalloimmunotherapy by rewiring innate immune responses and remodeling the tumor immune microenvironment, with clinically relevant potential further supported by its enhanced efficacy when combined with anti-PD-L1 checkpoint blockade.

## CRediT authorship contribution statement

**Guoyu Xia:** Conceptualization, Data curation, Formal analysis. **Chenyang Wang:** Data curation, Formal analysis. **Liyuan Peng:** Data curation, Formal analysis. **Weiyu Xing:** Data curation, Formal analysis. **Lulu Wang:** Conceptualization, Data curation. **Zhen Zheng:** Conceptualization, Data curation, Formal analysis.

## Declaration of competing interest

The authors declare that they have no known competing financial interests or personal relationships that could have appeared to influence the work reported in this paper.

## Data Availability

The data that support the findings of this study are available in the supplementary material of this article.
