# Supplementary material for: Programming Mn(II) coordination in self-assembling peptides amplifies mtDNA-driven STING signaling for potent antitumor immunity
Source: Mater Today Bio. 2026 Jun 18;39:103373. doi: 10.1016/j.mtbio.2026.103373 (PMC13315962; doi:10.1016/j.mtbio.2026.103373)
Supplement: Multimedia component 1 [file mmc1.docx]

***Supporting Information***

**Programming Mn(II) Coordination in Self-Assembling Peptides Amplifies mtDNA-Driven STING Signaling for Potent Antitumor Immunity**

Guoyu Xia^1^, Chenyang Wang^1^, Liyuan Peng^1^, Weiyu Xing^1^, Lulu Wang^1,*^, Zhen Zheng^1,*^

^1^Tianjin Key Laboratory on Technologies Enabling Development of Clinical Therapeutics and Diagnostics, School of Pharmacy, Tianjin Medical University, Tianjin 300070, China

*Corresponding authors:

wanglulu@tmu.edu.cn (Lulu Wang), zhengzhen90@tmu.edu.cn (Zhen Zheng).


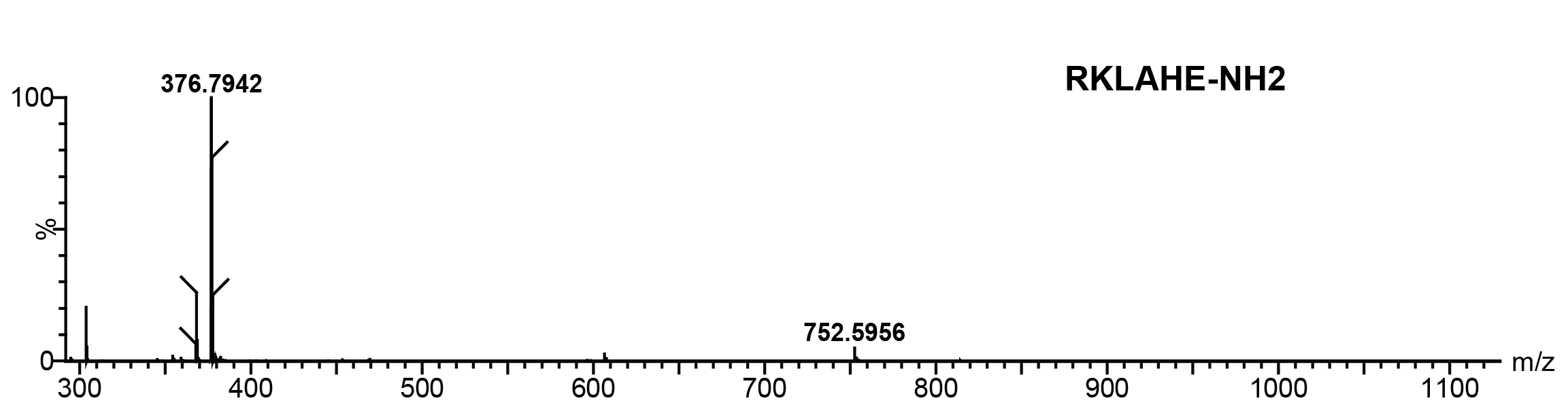


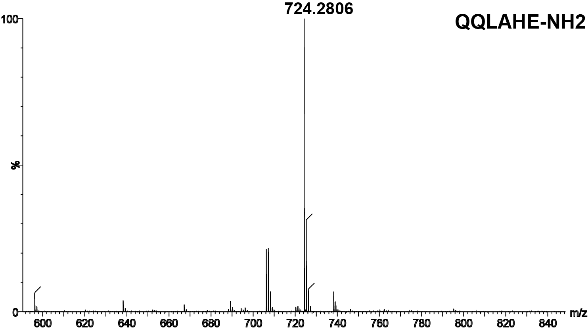

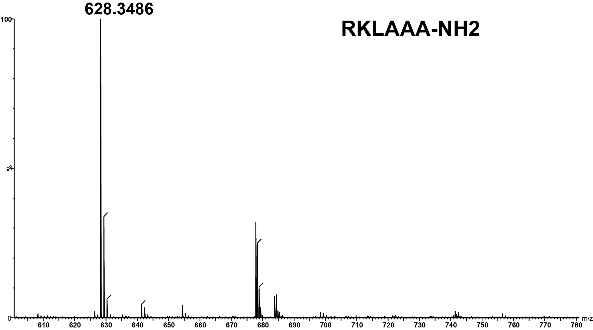


**Figure S1.** MS spectrum of RKLAHE–NH₂, QQLAHE-NH_2_, RKLAAA-NH_2_.
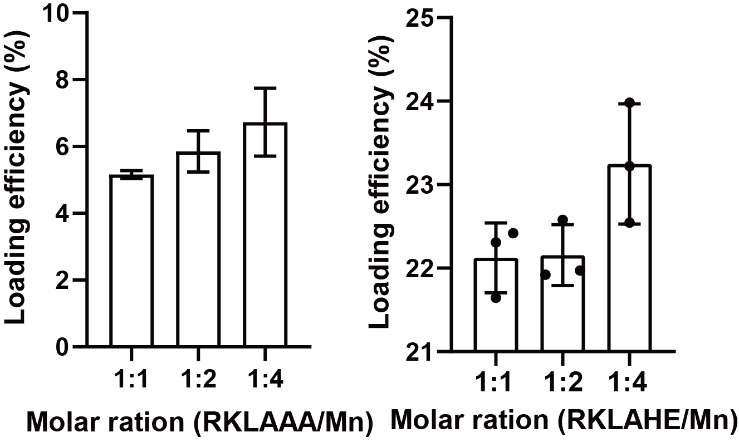


**Figure S2.** Mn²⁺ loading efficiencies of RKLAHE-Mn and RKLAAA-Mn formulations prepared at different initial peptide/Mn²⁺ feed molar ratios (1:1, 1:2, and 1:4), as quantified by ICP-MS. Data are presented as mean ± SEM (n = 3).


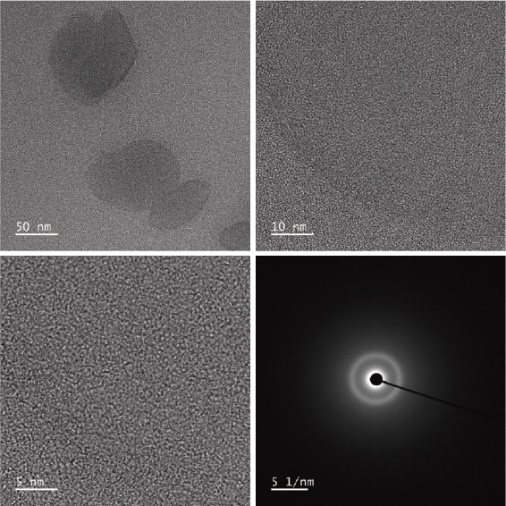


**Figure S3.** High resolution TEM image and selected area electron diffraction pattern of RKLAHE–Mn nanoassemblies.


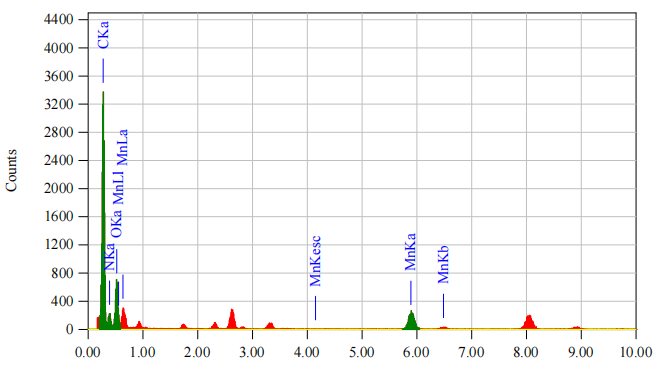


| **Element** | **(keV)** | **Mass%** | **Counts** | **Sigma** | **Atom%** | **Compound Mass%** |
| --- | --- | --- | --- | --- | --- | --- |
| C K (Ref.) | 0.277 | 83.14 | 13135.19 | 0.55 | 88.96 | 1.0000 |
| N K*! | 0.392 | 2.61 | 610.63 | 0.11 | 2.40 | 0.670 |
| O K*! | 0.525 | 9.33 | 2883.25 | 0.22 | 7.49 | 0.5110 |
| Mn L* | 5.894 | 4.92 | 2088.32 | 0.17 | 1.15 | 0.3724 |
| **Total** |  | 100.00 |  |  | 100.00 |  |

**Figure S4.** Quantitative elemental analysis of EDS mapping for RKLAHE–Mn, showing the relative atomic fractions of C, N, O, and Mn derived from the mapping dataset


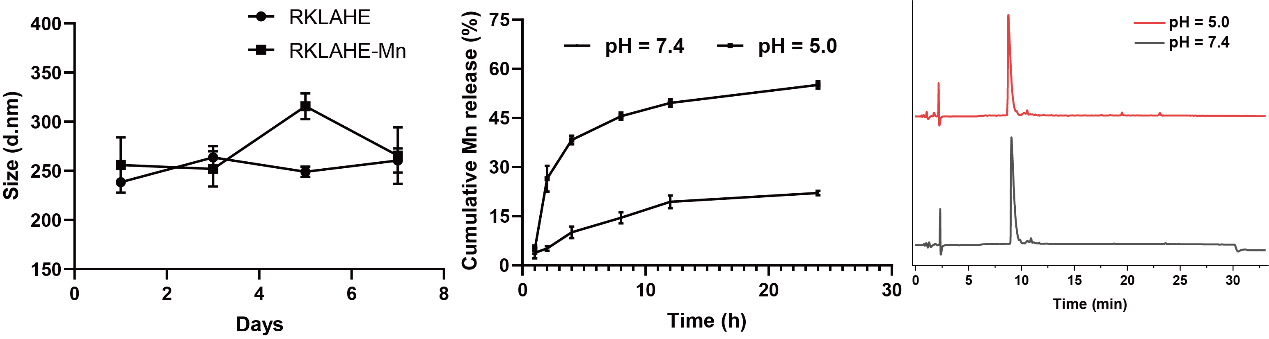


**Figure S5.** Serum stability of RKLAHE and RKLAHE–Mn, pH-dependent Mn release from RKLAHE–Mn, and HPLC analysis of RKLAHE after 24 h incubation.


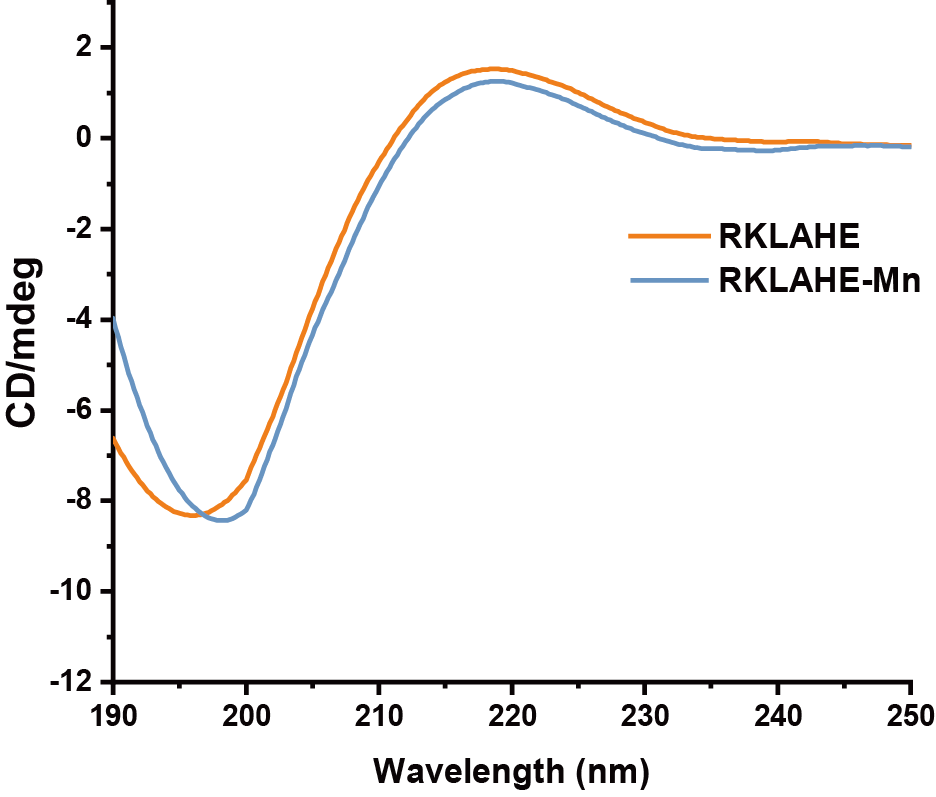


**Figure S6.** Circular dichroism spectra of RKLAHE and RKLAHE–Mn in aqueous solution.


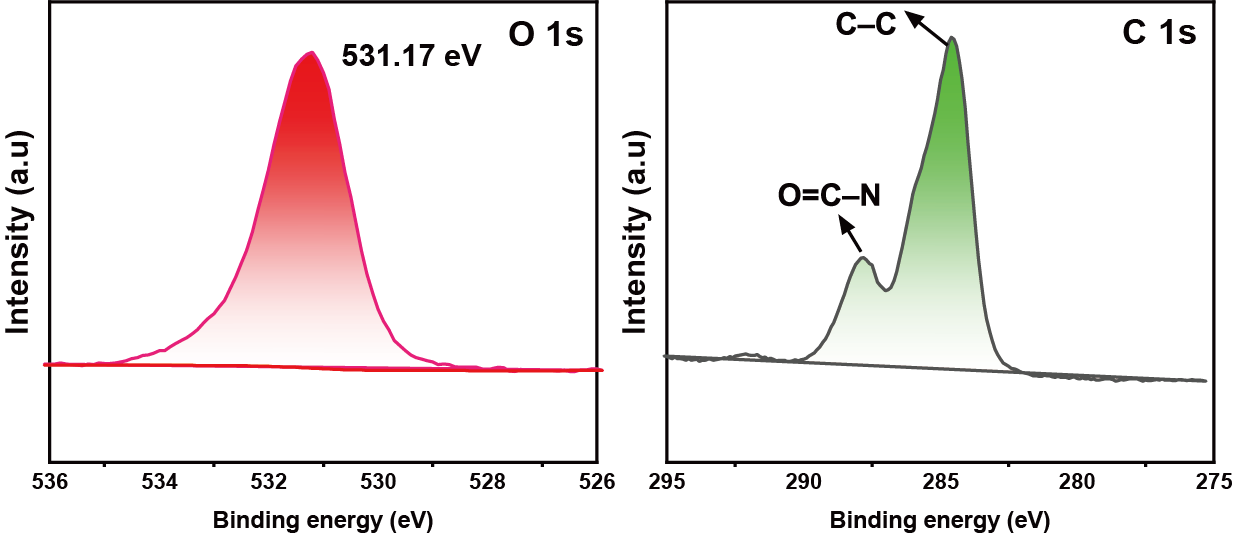


**Figure S7.** High-resolution O 1s and C 1s XPS spectra of RKLAHE-Mn.


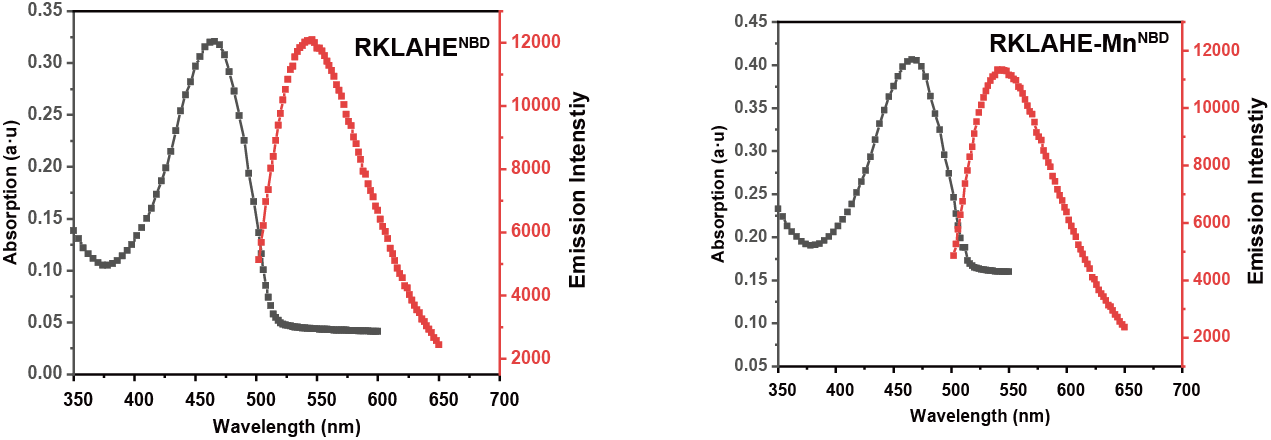


**Figure S8.** UV–vis absorption spectra and fluorescence emission spectra of RKLAHE NBD and RKLAHE–Mn NBD


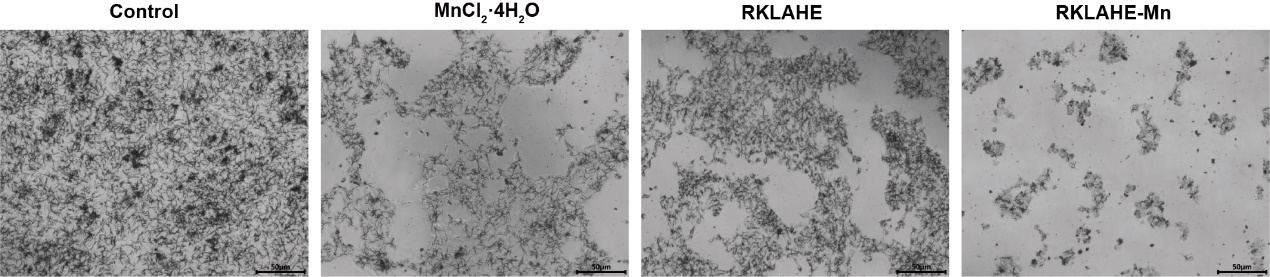


**Figure S9.** Representative bright field microscopy image of formazan crystals formed in the MTT assay after incubation.


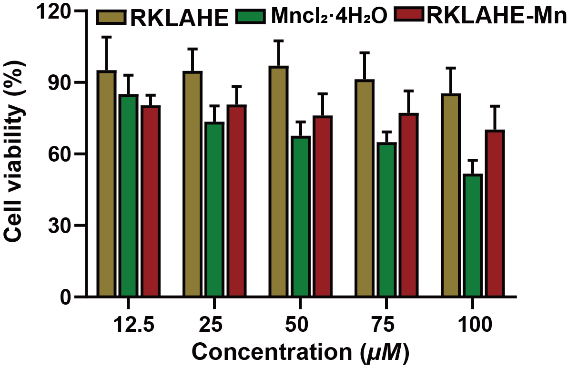


**Figure S10.** Cell viability of HUVEC cells after incubation with MnCl₂·4H₂O, RKLAHE, or RKLAHE–Mn at the indicated concentrations. Data are presented as mean ± SEM (n = 4).


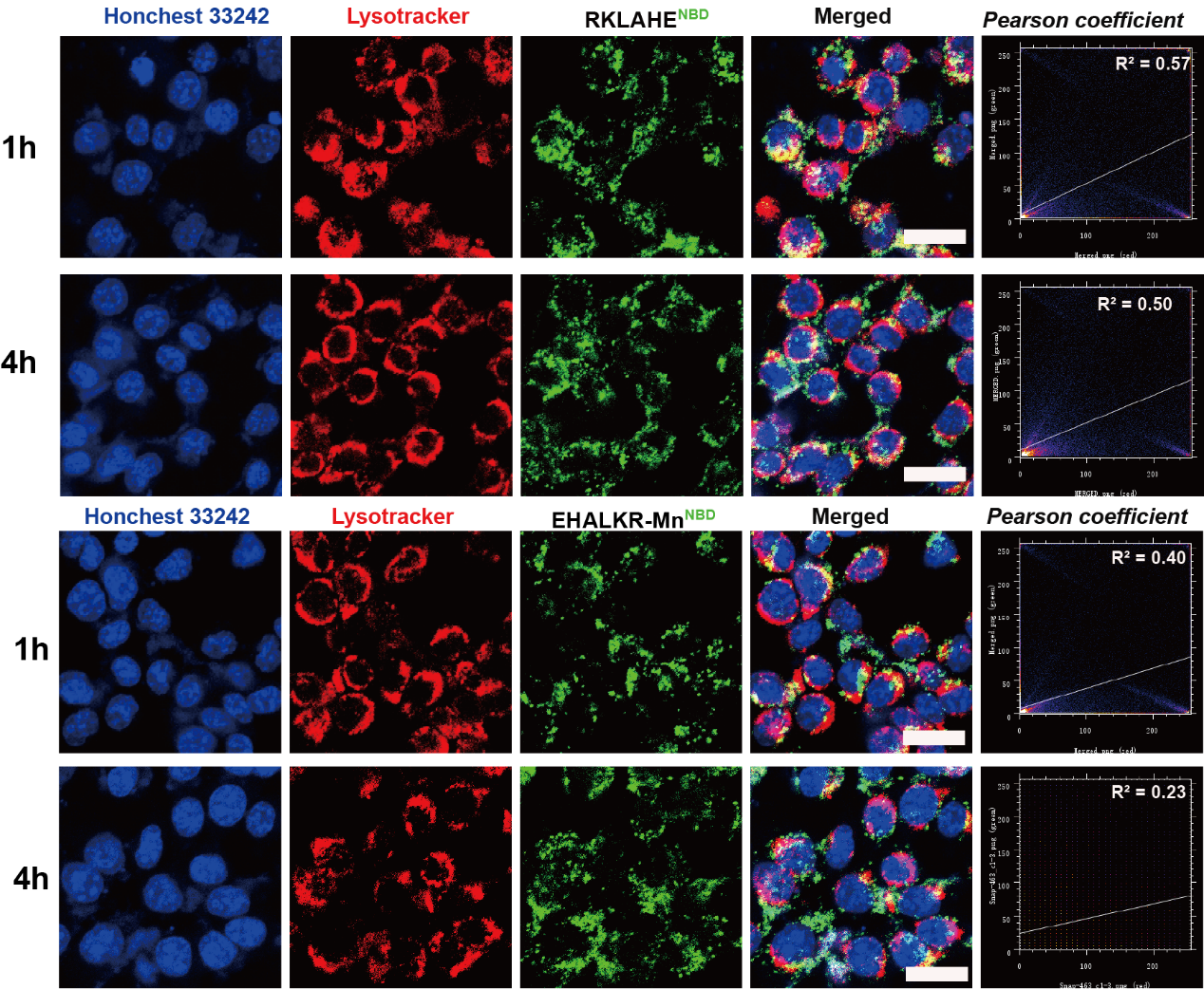


**Figure S11.** Confocal microscopy analysis of lysosomal colocalization of RKLAHE NBD and RKLAHE–Mn NBD in 4T1 cells after incubation for 1 h and 4 h. Lysosomes were stained with LysoTracker, nuclei were counterstained with Hoechst 33342, and colocalization was quantified using Pearson correlation analysis


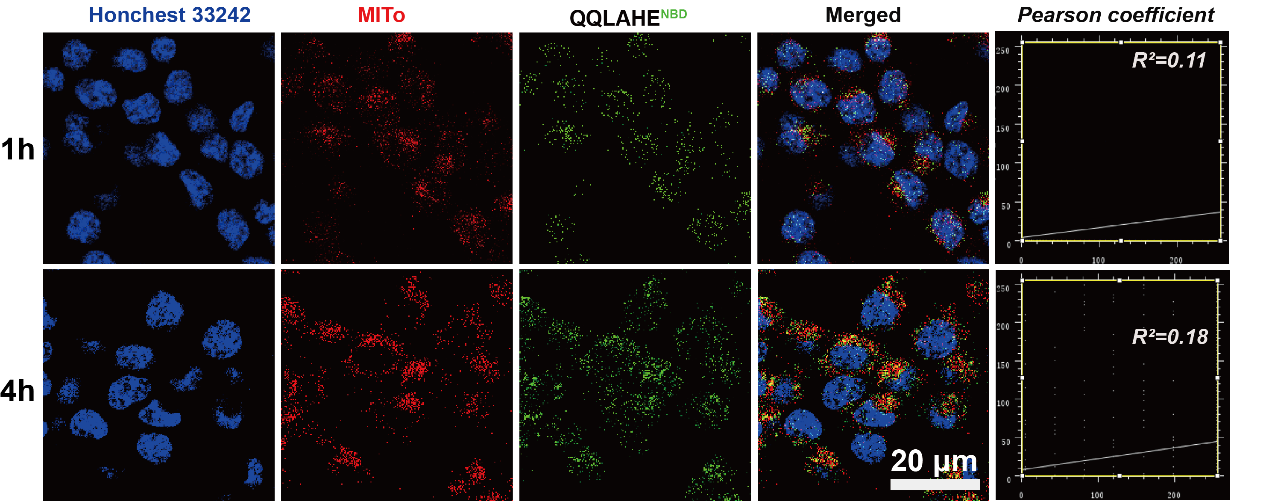


**Figure S12.** Mitochondrial colocalization analysis of charge-neutralized QQLAHE^NBD^_._

**Figure S13**. Mitochondrial Mn content after different treatment. Mn in isolated mitochondrial fractions was quantified by ICP-MS and normalized to 10⁶ cells. Data are presented as mean ± SEM (n = 3). ***P < 0.001.**


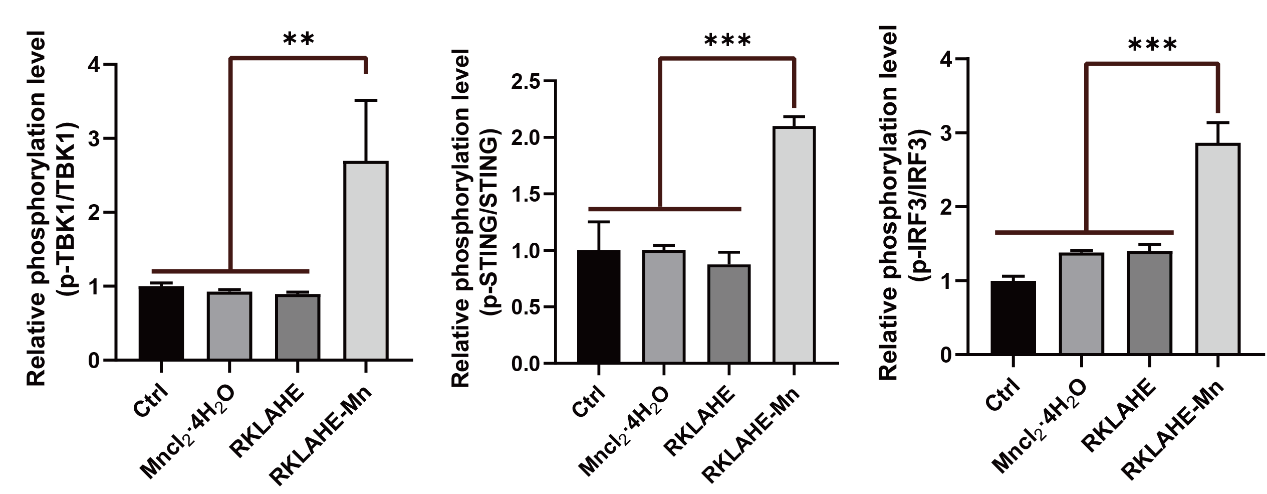


**Figure S14**. Quantitative analysis of the relative phosphorylation levels of p-STING/STING, p-TBK1/TBK1, and p-IRF3/IRF3 depicted in Figure 3C.


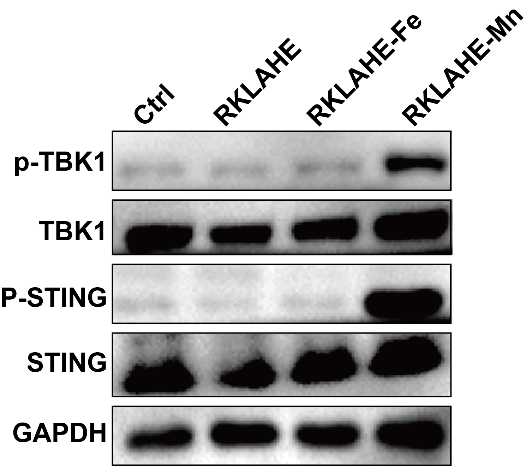


**Figure 15**. Western blot images after treatment with RKLAHE–Mn, RKLAHE–Fe, RKLAHE, control group.


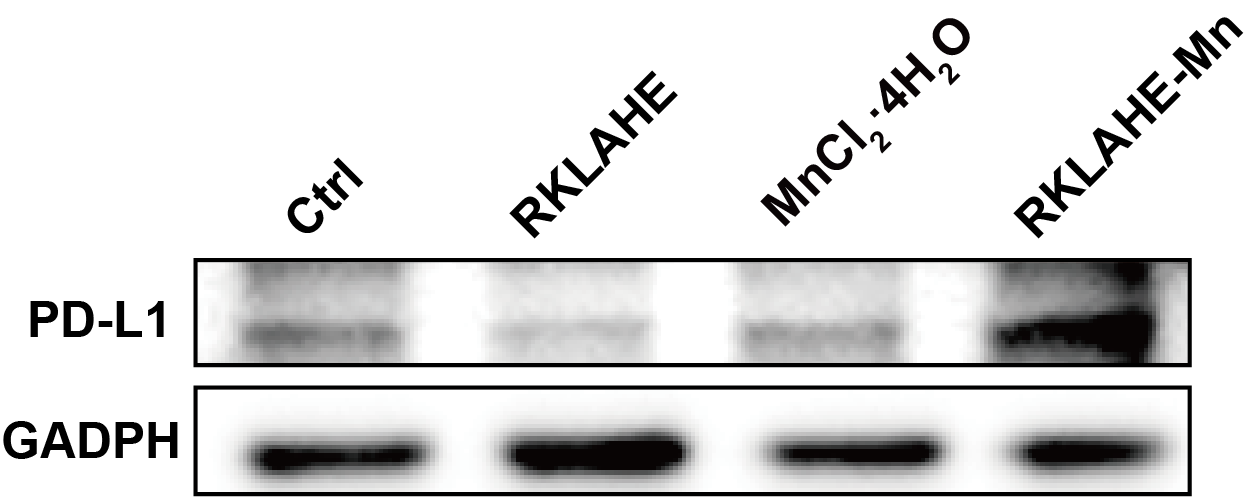


**Figure S16**. PD-L1 Western blot images after treatment with RKLAHE–Mn, Mncl₂ 4H₂O , RKLAHE, control group.


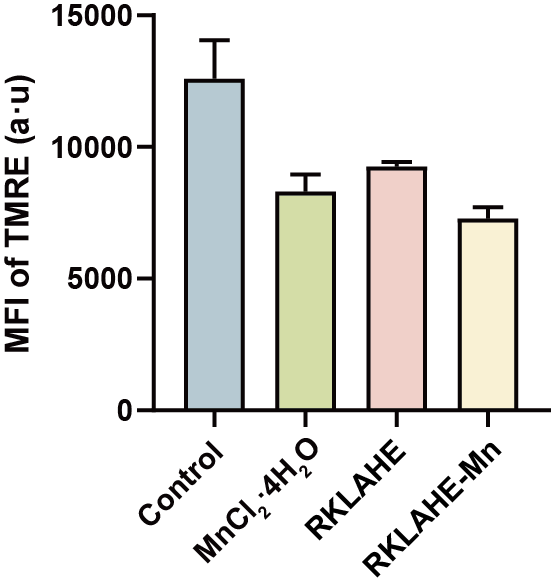


**Figure S17.** Quantification of mitochondrial membrane potential in 4T1 cells after different treatments, determined by TMRE staining. Data are presented as mean ± SEM (n = 3).


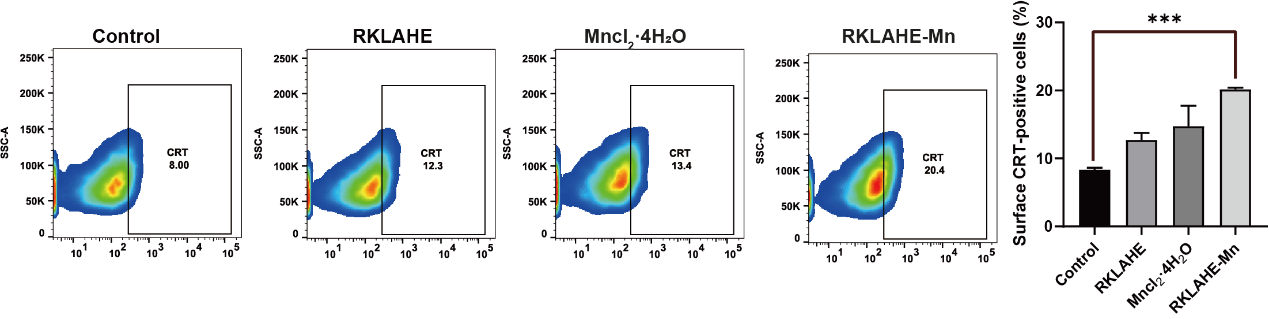


**Figure S18**. Flow cytometric analysis of surface CRT exposure after 24 h treatment. Data are presented as mean ± SEM (n = 3).


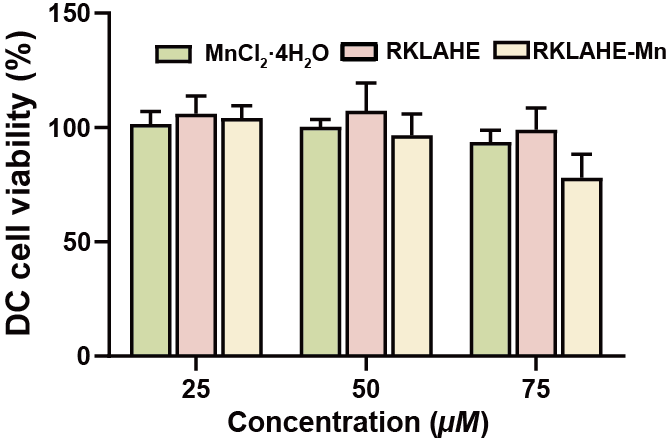


**Figure S19.** Cell viability of DC2.4 cells after incubation with MnCl₂·4H₂O, RKLAHE, or RKLAHE–Mn at the indicated concentrations. Data are presented as mean ± SEM (n = 4).


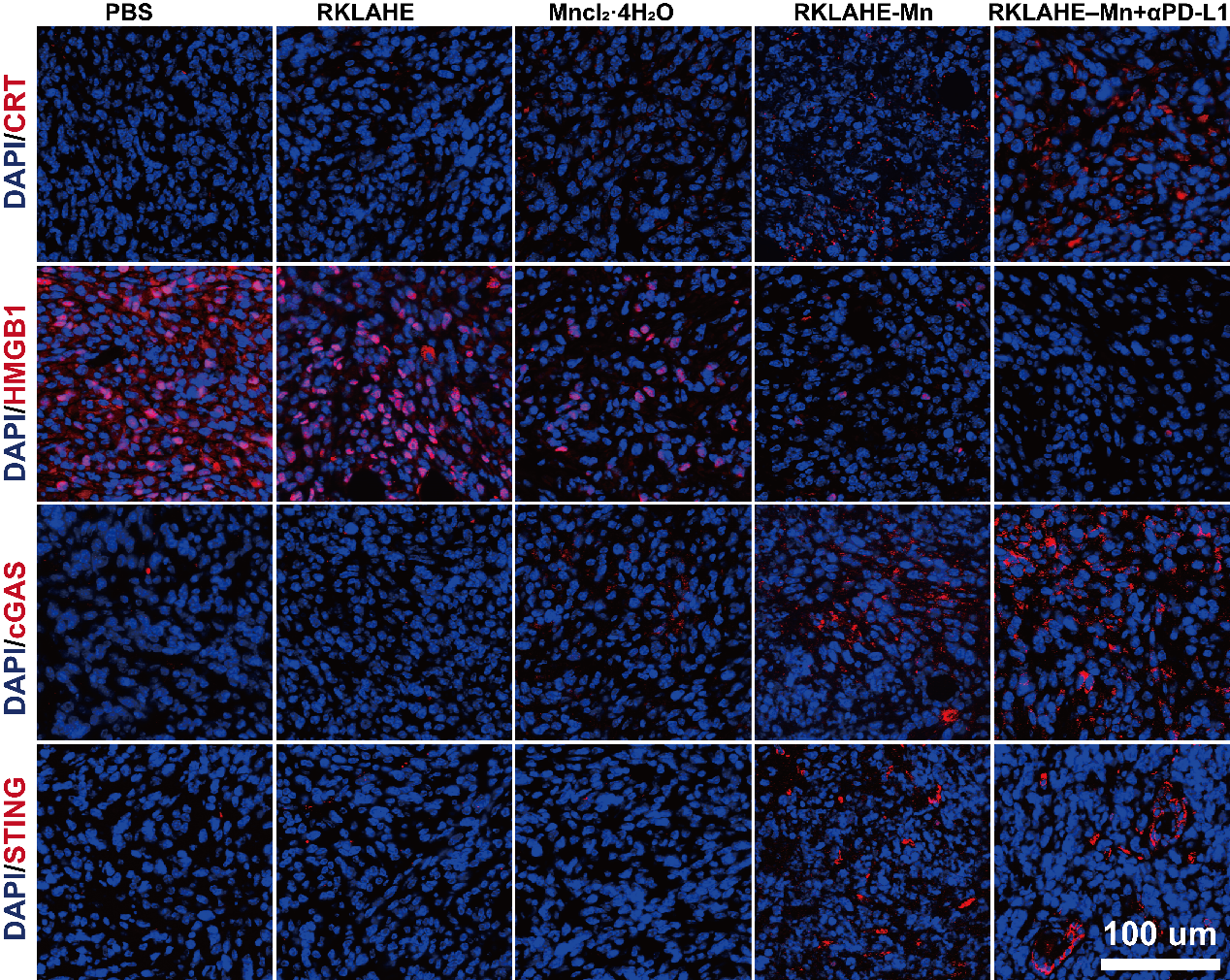


**Figure S20** Immunofluorescence staining of CRT, HMGB1, cGAS, and STING in tumor sections from mice receiving the indicated treatments.


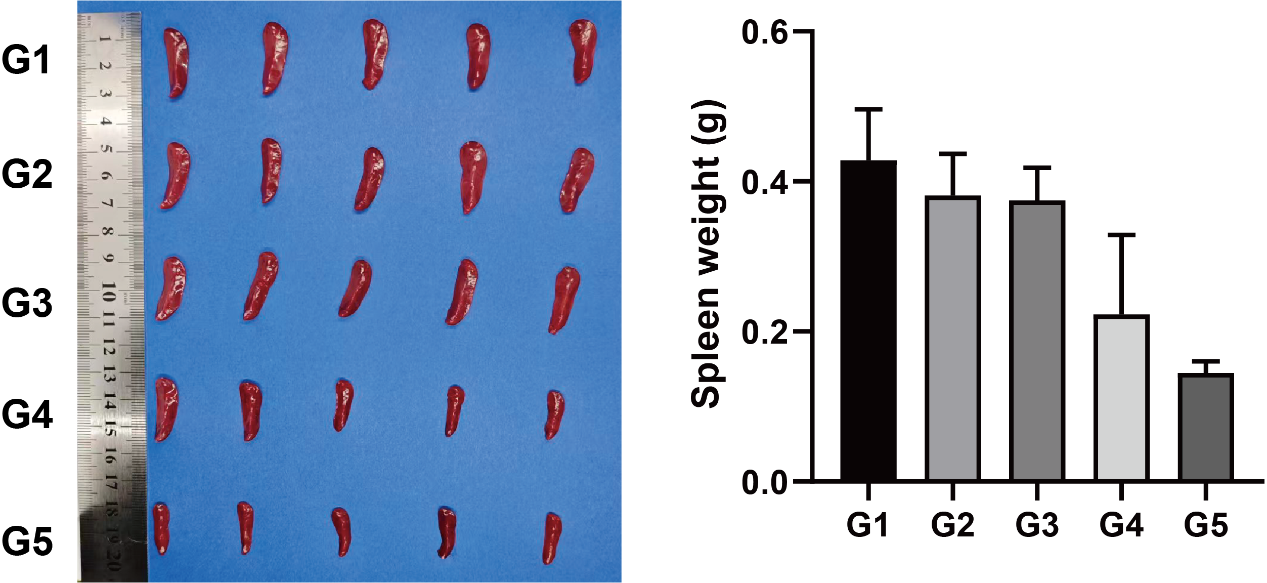


**Figure S21.** Representative photographs of spleens collected at the end of treatment and corresponding spleen weight statistics. Data are presented as mean ± SEM (n = 5).


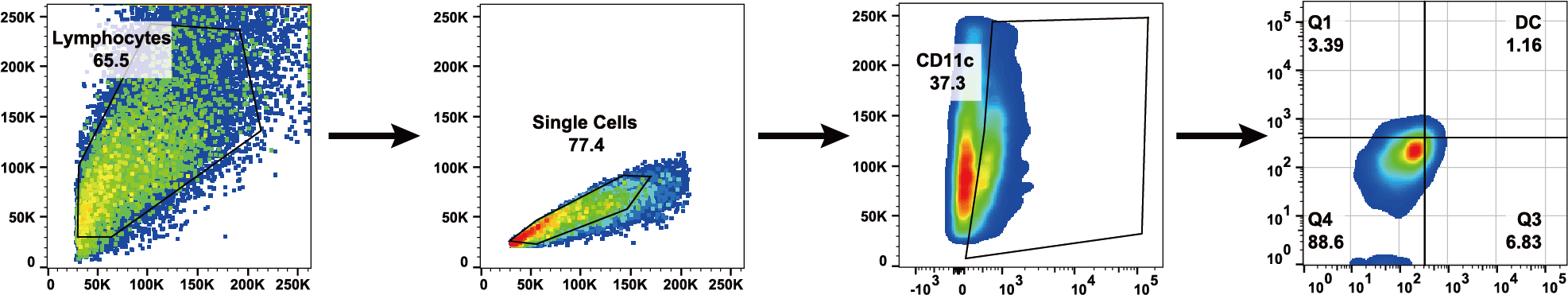


**Figure S22**. The gating strategy used to determine the percentages of mature dendritic cells (CD80⁺CD86⁺ within CD11c⁺) in Figure 6B.


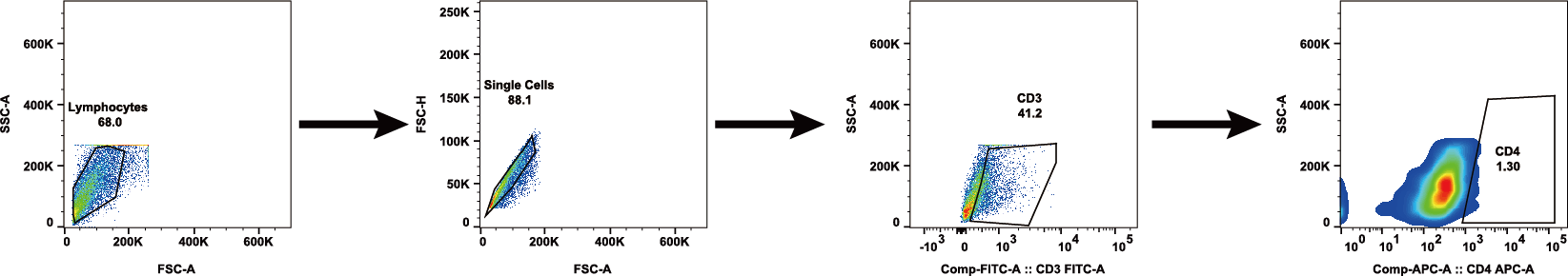


**Figure S23**.The gating strategy used to determine the percentages of intratumoral CD8⁺ T cells (CD8⁺ within CD3⁺) in Figure 6C.


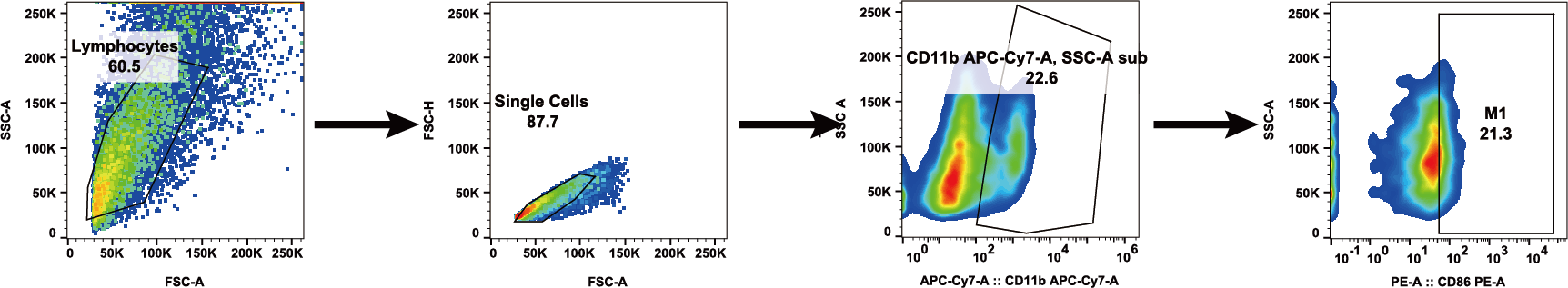


**Figure S24**.The gating strategy used to determine the percentages of M1-like macrophages (CD86⁺ within CD11b⁺) in Figure 6D.


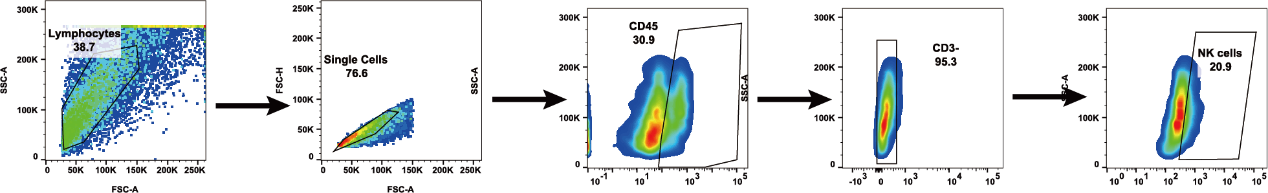


**Figure S25**.The gating strategy used to determine the percentages of NK cells (NKp46⁺ within CD45⁺CD3⁻) in Figure 6E.


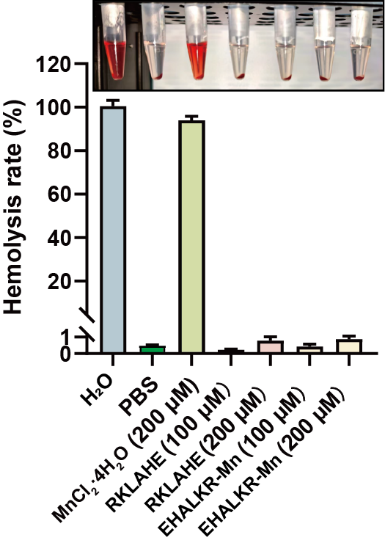


**Figure S26.** Hemolysis assessment of red blood cells incubated with H₂O, PBS, MnCl₂·4H₂O, RKLAHE, and RKLAHE–Mn at the indicated concentrations. Representative photographs of the supernatants are shown. Data are presented as mean ± SEM (n = 3).


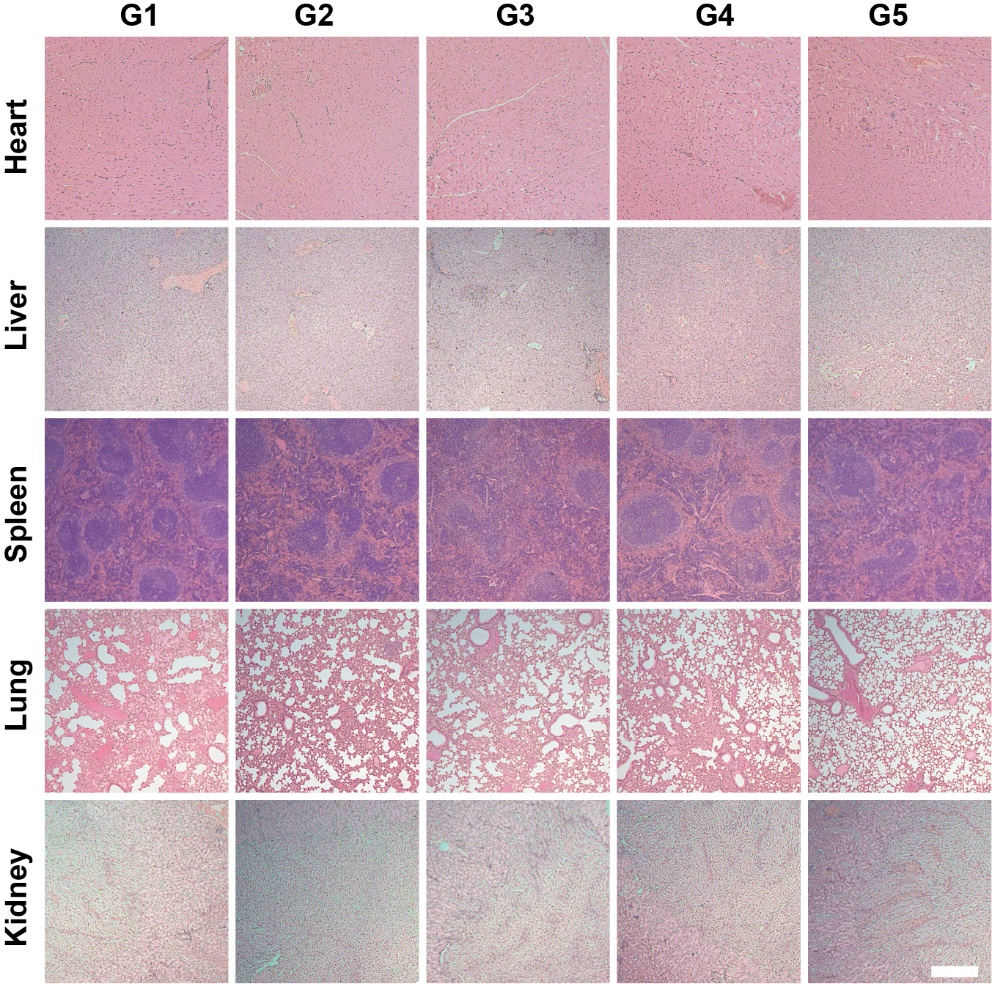


**Figure S27.** Representative H&E stained histological sections of major organs (heart, liver, spleen, lung, and kidney) collected after treatment. Scale bar: 200 μm.
